# Indian Craniometric Variability and Affinities

**DOI:** 10.1155/2013/836738

**Published:** 2013-12-24

**Authors:** Pathmanathan Raghavan, David Bulbeck, Gayathiri Pathmanathan, Suresh Kanta Rathee

**Affiliations:** ^1^School of Archaeology and Anthropology, College of Arts and Social Sciences, The Australian National University, Canberra, ACT 0200, Australia; ^2^Department of Archaeology and Natural History, College of Asia and the Pacific, The Australian National University, Canberra, ACT 0200, Australia; ^3^Department of Anthropology, Panjab University, Chandigarh IN-CH 160001, India; ^4^Department of Anatomy, Post Graduate Institute of Medical Sciences, Rohtak IIM-R 124001, India

## Abstract

Recently published craniometric and genetic studies indicate a predominantly indigenous ancestry of Indian populations. We address this issue with a fuller coverage of Indian craniometrics than any done before. We analyse metrical variability within Indian series, Indians' sexual dimorphism, differences between northern and southern Indians, index-based differences of Indian males from other series, and Indians' multivariate affinities. The relationship between a variable's magnitude and its variability is log-linear. This relationship is strengthened by excluding cranial fractions and series with a sample size less than 30. Male crania are typically larger than female crania, but there are also shape differences. Northern Indians differ from southern Indians in various features including narrower orbits and less pronounced medial protrusion of the orbits. Indians resemble Veddas in having small crania and similar cranial shape. Indians' wider geographic affinities lie with “Caucasoid” populations to the northwest, particularly affecting northern Indians. The latter finding is confirmed from shape-based Mahalanobis-*D* distances calculated for the best sampled male and female series. Demonstration of a distinctive South Asian craniometric profile and the intermediate status of northern Indians between southern Indians and populations northwest of India confirm the predominantly indigenous ancestry of northern and especially southern Indians.

## 1. Introduction

This paper analyses the metrical variability of human crania within the Indian subcontinent and uses the results to inform a univariate, bivariate, and multivariate comparison of Indian and other crania. India's importance for understanding anatomically modern human origins is widely recognised: India has the highest genetic diversity of any continental region after Africa [1] and is generally regarded as the major dispersal centre for *Homo sapiens* following our exodus from Africa [2]. Yet India has been comparatively neglected in human craniometric studies, for instance, in being excluded from the global survey of modern human crania undertaken by Howells [3]. Studies that have included Indian crania have been restricted to specimens held in overseas collections [4–10]. In addition, most of these studies have been based on a limited set of measurements, and none of them combine a presentation of descriptive statistics with a large-scale multivariate analysis of the data. The motivation of our paper is to explain Indians' craniometric affinities in the context of a thoroughgoing statistical description of Indian crania (see Supplementary Material available online at http://dx.doi.org/10.1155/2013/836738).

Previously undertaken multivariate studies are consistent in pointing to a similarity between crania from India and from surrounding locations. Stock et al. found that both northern and southern Indian crania cluster tightly together. Closest to Indians are crania from Afghanistan and Iran, the Andaman Islands, Sri Lanka (Veddas and to a lesser degree Sinhalese), and at a greater remove southwest Asia [9]. Similarly, Wright found that his Indian sample clusters with Andaman Islanders, the latter being otherwise close to southwest Asians and Egyptians [10]. Brace et al. found that northern and southern Indians constitute a discrete cluster, along with Sri Lanka crania. These South Asians cluster with Europeans if Andamanese are excluded from analysis, or with Andamanese if Europeans are excluded from analysis [6]. The impression these studies give is that northern and southern Indians are very similar in their craniometrics, with secondary affinities to Sri Lanka, Andamanese, and southwest Asian crania, regardless of whether differences between populations in cranial size are controlled for [6, 9] or not [10].

Until the mid-2000s, theories on the population history of India distinguished between indigenous and immigrant strata [7, 11–14]. The indigenous stratum supposedly consisted of foragers of “Australoid” or “proto-Australoid” racial affinity, with the “Veddoids” (represented by the Veddas) sometimes recognised as a distinct component. The foragers either survived into recent times as enclaves or else were absorbed during the Holocene expansion of farming populations into India from Central Asia and/or the Mediterranean. Two separate demographic expansions were recognised, one leading to Dravidian speakers in the south and the other to Indo-European (Indo-Aryan) speakers in the north. An additional incursion of farming populations, restricted to northeast India, involved Munda (Austro-Asiatic) speakers with Southeast Asian (“Mongoloid”) affinities.

The recent accumulation of genetic evidence for the Late Pleistocene origin of *Homo sapiens* in Africa has had two main implications for understanding India's population history. The first is to reinterpret India's indigenous stratum as the first wave of *Homo sapiens* colonists en route from Africa to Eurasia and Australia. The second main implication is to cast doubt on the concept of a Dravidian migration and to interpret any Central Asian genetic affinities in southern India as a knock-on effect following the Indo-European immigration into northern India [1, 15]. In the same vein, Reich et al. recognise a distinction between “Ancestral North Indian” and “Ancestral South Indian” complexes. The former is closely related to Middle East, Central Asian, and European populations whereas the latter has no demonstrable similarities with other Eurasians. Within the Indian subcontinent, Indo-Aryan speakers have predominantly “Ancestral North Indian” ancestry and Dravidian speakers predominantly “Ancestral South Indian” ancestry, while the Onge of the Andaman Islands have retained undiluted “Ancestral South Indian” ancestry [16].

Our paper compares Indian crania with the series recorded by Howells [17] on a large suite of craniometric measurements, to test three hypotheses on Indian affinities arising from recent genetic studies. The first hypothesis is that northern and southern India crania can be more clearly distinguished from each other than earlier craniometric studies have indicated. The second hypothesis is that northern Indian crania will show affinities to Howells' Egyptian and European crania. The third hypothesis is that southern Indian crania will show affinities with Andaman Islander rather than Egyptian and European crania.

## 2. Materials and Methods

**Figure 1:**
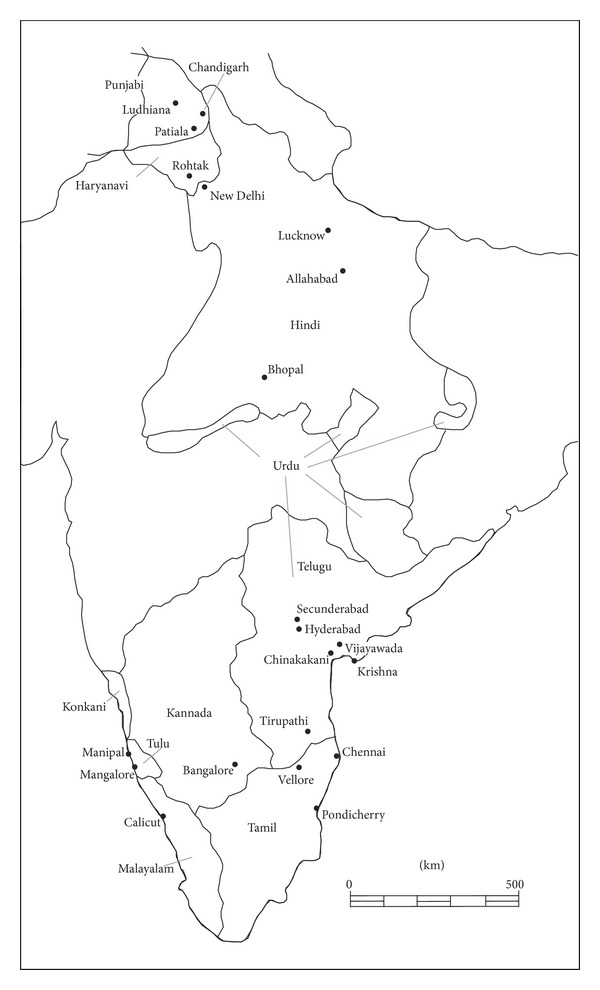
Distribution of Indian languages covered in this study and locations of holding institutions.

Between 2001 and 2005, the first author measured over 1,300 adult crania held in anatomical institutions across India, along with a small number in Adelaide, Australia. Twelve ethnic groups are represented amongst the cranial collections but two of them, the Coorg and Bengalis, are unfortunately available only as very small sample sizes. Also, the first author did not come across crania from many of the “tribal groups” that would be of potential interest for our study, groups such as the Munda, Santhal, Yanadi, and Irula.  Table 1 and Figure 1 present the language, language distribution [18, 19], geographic location, and holding institutions of the ten series that could be included in this study.

Most of the measured crania were obtained from medical dissections of adults of known language affiliation, with smaller numbers donated by collectors or recovered from historical gravesite excavations. Over 90 percent are of known adult status and sex, as recorded in the mortuary registers, and some come from named individuals. In addition the first author, often accompanied by senior curatorial staff, inspected the crania for their degree of dental development and cranial suture closure to confirm their adult status, as well as their general size and the robustness of their mastoid process, supraorbital region, and nuchal musculature to confirm their recorded sex. This familiarisation with the morphological variation shown by adult males and females of each Indian series allowed the first author to sex the adult crania of unrecorded sex, assisted by documentation of the pelvis and other postcranial bones where these were available.

Crania whose measurements appeared to be affected by recorded signs of pathology were excluded.

The first author took all 47 craniometric variables defined by Howells [3] excluding his radii (Table 2). He transcribed his measurements and specimen documentation to an Excel spreadsheet, against which the second author checked the original records. The second author also performed logical checks on the data, including calculation of 17 indices (Table 2) that reflect main aspects of cranial shape (wherever the numerator and denominator measurements were both available). Any noted inconsistencies were resolved through mutual agreement. The Supplementary Material presents the sample sizes, means, standard deviations, and ranges for the measurements and indices of the ten series included in this study.

Six analyses are presented in this paper, making use of our large craniometric database, which allows us to exclude any specimens missing the analysed variable or variables. The first deals with intraseries variability, considering both the samples' standard deviation and their range (the difference between the largest and smallest value within the sample). The second analysis focuses on sexual dimorphism and the third investigates craniometric variation within India. Determination of statistical significance in these analyses is based on the weighted Simes test [20]. The fourth analysis compares male average measurements and indices of South Asians, including the Veddas of Sri Lanka based on previously published data [5, 7], with those from other parts of the world. Males rather than females are chosen here because they are better sampled. The fifth and sixth analyses employ multivariate techniques to compare the six best sampled Indian series with the Howells series (males and females). The first of these analyses is a principal components analysis [21], and the second is based on Mahalanobis-*D* distances calculated from Mosimann indices [22] which are widely used in osteometric studies where the focus of attention is shape [23–26]. All multivariate analyses were undertaken using XLSTAT.

A note of caution arises from the decision by Howells [3] to substitute missing variables in the crania he measured with the average measurement from the series concerned. One obvious effect is to artificially increase the sample size for at least some variables and artificially decrease these variables' standard deviations. These points render Howells' data inappropriate for intraseries variability analysis. As for Howells' estimates of his series' means, included in our fourth analysis, the mean values themselves will not be affected, and therefore indices calculated from his series' means are also not affected. The effects on the fifth and sixth analyses are unknown but probably slight because Howells focused on crania with the great majority or all of their measurements intact.

## 3. Results

### 3.1. Analysis of Variability

Variability, as measured both with the standard deviation and the range, tends to increase as the mean increases, for all variables and across all series. However, the standard deviation and range increase at a far slower rate than that of the mean. While the linear relationship between the mean and the standard deviation is moderate (Pearson's *r* = 0.74), the slope of the best-fit line at 0.03 is flat. Similarly, Pearson's *r* for the linear relationship between the mean and the range is 0.70, while the slope of the best-fit line is merely 0.14 (Tables 3 and 4).

The association between the mean and variability measures improves markedly by the following two steps, although the slope of the best-fit lines remains flat. The first step is to exclude Howells' parietal, frontal, and occipital fractions on the basis of their excessive variability. Even though these cranial fractions (by definition) are smaller than their respective cranial chords, it is the cranial fractions that generally have the larger standard deviation (Supplementary Tables S3 and S4). When fractions are excluded, Pearson's *r* correlation coefficient increases to 0.81 comparing means and standard deviations and to 0.75 comparing means and ranges (Tables 3 and 4). The second step is to exclude sample sizes less than 30, based on the rule of thumb that 30 is a sufficiently large sample size to reliably estimate the main parameters of a population [27]. When the smaller samples are excluded, Pearson's *r* correlation coefficient increases to 0.89 comparing means and standard deviations and to 0.85 comparing means and ranges.

The coefficient of variation, or mean divided by the standard deviation, widely used in biostatistical analyses [28] has been critiqued as not applicable in comparing one variable with another [29]. As the present analysis shows, in the case of craniometric variables, the standard deviation increases with the mean albeit at a far slower rate. In fact, the relationship of the standard deviation and the range to the mean is log-linear rather than linear, as shown by expressing these variables as logarithmic values. Following this transformation to the variables, Pearson's *r* following this transformation to the variables, is always higher than was the case with the untransformed variables. Also, the slope of the best-fit line is always above 0.4, close to the 0.5 value that would reflect equal rates of increase between the variables being compared (Tables 3 and 4).

The preceding analysis suggests that the range is just as useful as the standard deviation in systematically charting variability within a cranial series. While the range has a poor reputation for being affected by extreme cases, the point being made here is that extreme cases can be expected for any well-sampled series. For instance, if we consider vault length (GOL) for males and females with a sample size of at least 30, the smallest male value is always less than 162 mm and the largest male value is always greater than 190 mm, while the smallest female value is always less than 159 mm and the largest female value is always greater than 182 mm (Supplementary Table S1). Thus, any temptation to “cleanse” a series by trimming it of specimens with extreme measurements—for instance, measurements more than two standard deviations from the mean—should be avoided, as it would impose an artificial homogeneity on the series. However, where particular variables depart from the general pattern shown by the other variables, as observed here with cranial fractions, these should be removed from analysis as their heightened variability is likely to be an artefact of measurement uncertainty.

Does the intraseries variability noted here primarily reflect differences in size, distinguishing crania with consistently large measurements from crania with consistently smaller measurements? If so, we may expect only a weak correlation between the means of the main indices and their variability. The relationship between the index mean and its variability is difficult to discern when indices are considered as a single set. For instance, Pearson's *r* for all indices for all series, comparing the index means and standard deviations, is low, at 0.17. However, this lack of a clear positive correlation is entirely due to extreme variability of the two indices that reflect protrusion of the nasal bones (DKB:NDS and WNB:SIS in Supplementary Table S12). (While intraseries variability of the nasodacryal index (DKB:NDS) has not previously been investigated, the high variability of the simotic index (WNB:SIS) has already been noted [5, 8].) When these two indices are excluded, index means show a moderate positive correlation with standard deviations and ranges (Table 5), as high as 0.78 (log-transformed index and standard deviation for all series with a sample size of at least 30). Generally speaking, indices resemble measurements in the degree to which variability scales with mean values, as would be consistent with considerable intraseries shape variability.

The extent and ubiquity of shape variation within Indian cranial series can be shown by considering index ranges (Supplementary Tables S1 to S10) in terms of the standard index categories used in physical anthropology [30]. All series except the Konkani include both hyperdolichocephalic crania with a cranial index less than 65 and brachycephalic crania with a cranial index above 80. All series other than the Konkani include chamaecranic individuals with a vault length-height index less than 70 and hyperhypsicranic individuals with a vault length-height index above 80. Similarly, the upper facial index ranges from hypereuryenic individuals (index less than 45) to lepten individuals (index above 55) in every series other than the Urdu. In every series, the orbital index ranges between chaemoconchic (less than 76) and hypsiconchic (above 85), and the nasal index ranges between leptorrhine (less than 47) and hyperchamaerrhine (above 58). Similarly, the frontal curvature index breakpoints proposed by Larnach and Macintosh [31] do not begin to capture the variability recorded for Indians. Every series includes individuals with very receding frontals as shown by an FRC:FRS index less than 21 and individuals with very bulging frontals as shown by an index above 27.

Although many of the following comparisons in this paper focus on series means, the results should not be interpreted in a typological sense, given the demonstration of how variable crania are within any Indian series.

### 3.2. Sexual Dimorphism

**Figure 2:**
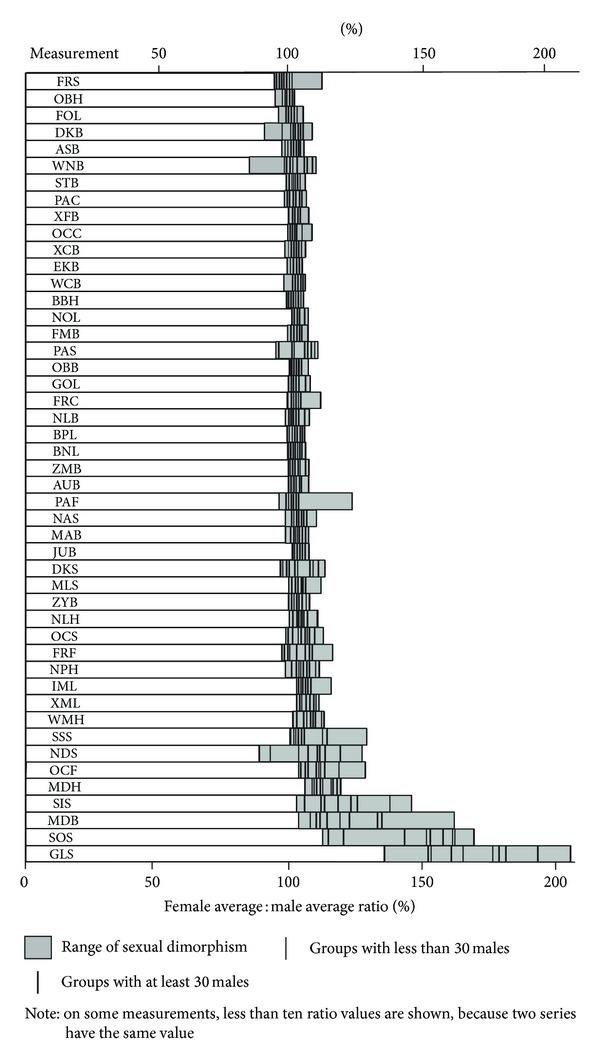
Sexual dimorphism of the ten Indian series for the Howells measurements, arranged in approximate ascending order.

The generally larger size of male compared to female crania, well established for populations worldwide, applies to Indians too. One way to illustrate this pattern is to divide the male average by the female average for the ten recorded Indian series, for each measurement, and present the resulting ratios as percentages (Figure 2). There are a few measurements where the male average is proportionately much larger than the female average, notably glabella subtense (GLS, around 150–200%, depending on series), supraorbital subtense (SOS, around 120–170%), and the dimensions of the mastoid process (MDB and MDH, around 110–150%). At the other end of the scale, some measurements show minimal sexual dimorphism, notably foramen magnum length (FOL), orbital height (OBH), and frontal subtense (FRS). By and large, however, there is a tendency for male averages to cluster at around 110% of the corresponding female averages.

Accordingly, the size of the difference between male and female averages largely reflects whether the measurement is big or small. If we subtract the female from the male average and correlate the result with the male average, excluding fractions and series with a sample size of less than 30 for both males and females, we find a Pearson's *r* correlation coefficient of 0.773. (Investigation of the correlation between these variables after log transformation is not possible because, as shown in Figure 2, the female average subtracted from the male average occasionally yields zero or a negative number, neither of which can be log-transformed.)

Another observation to be inferred is that the measurements with the greatest sexual dimorphism, as reflected by the average female : male ratio, also tend to be small (GLS to NLH in Figure 2). Small measurements are also distinguished from large measurements by a greater standard deviation in relation to the mean (Table 3), which makes the proportionate relationship between the male and female means an unreliable predictor of whether or not there is a statistically significant difference between the male and female means. This is demonstrated in Table 6, which presents an analysis in terms of the series for which males are significantly larger than females (one-tailed Student's *t*-test, *P* set at 0.05 or a smaller number as required by the weighted Simes test).

At one extreme are six measurements significantly larger for males than females in all ten Indian series. At the other extreme are four measurements that are not significantly larger for males than females in any of the series (including FRS, which is actually larger for females than males in all Indian series with a male sample size of at least 30). In between are 16 measurements significantly larger for males than females as long as the male sample size is at least 30; 11 measurements significantly larger for the clear majority of series with a male sample size of at least 30; and eight measurements with weak sexual dimorphism. These intermediate cases include all four measurements (GLS, SOS, MDB and SIS) with the most pronounced sexual dimorphism based on the proportionate comparison of the male and female means.

The ordering of series in Table 6 shows the importance of adequate sample size to identify sexual dimorphism in human cranial measurements. The Tulu, Haryanavi, Telugu, Punjabis, Kannada, Tamils, and Hindis, with a minimum sample size of at least 30 for males (and 18 for either sex), all show males to be significantly larger than females for around 60–80% of measurements. In contrast, the Malayalam, Konkani, and Urdu, represented by smaller sample sizes, can be shown to be sexually dimorphic for just 20–30% of measurements.

For most indices, males and females from the same Indian series show very similar average values (Supplementary Tables S1 to S12), especially where sample size is large enough to be reliable (as for the bottom seven series in Table 6). However, there are several indices where males and females consistently differ. Average frontal curvature index (FRC:FRS) is larger for females than males in the same series, to a degree that is statistically significant as long as male sample size is at least 30. This accords with the recognition that male frontals tend to slant back more strongly compared to females' more rounded frontals [32]. Females exceed males from the same series in their average orbital index (OBB:OBH), to a statistically significant degree in the case of Hindis, Kannada, Tamils, Telugu, Tulu, and Konkani. This is consistent with the recognition that females tend to have a more rounded upper orbital margin than males [33]. On the other hand, males' XCB:ZYB index consistently exceeds that of females in the same series, significantly so for Hindis, Kannada, Tamils, Tulu, and Urdu, related to the presence of a wider zygomatic arch as a male marker for the human skull [32]. Finally, males tend to have a more prominent nasal skeleton than females from the same series. This is reflected by males' significantly larger nasodacryal or DKB:NDS index for Hindis, Haryanavis, and Telugu and larger simotic (WNB:SIS) index for Hindis, Tamils, and Telugu. These instances of sexual dimorphism in cranial shape suggest a potential shortcoming in multivariate statistical studies [6, 9] that pool males and females in the same analysis, attempting to accommodate sexual dimorphism by simply compensating for cranial size.

### 3.3. Craniometric Variability within India

For most measurements and indices, when the means are considered, consistent differences between northern and southern Indians, or between Indo-Aryan and Dravidian speakers, are difficult to discern. For instance, the range of means for cranial length is 176.5–181.9 mm for northern Indian males and 176.1–181.9 mm for Indo-Aryan males, which overlap extensively with the range of means of 173.0–178.6 mm for southern Indian males and 173.0–178.5 mm for Dravidian males (Supplementary Table S1). Similarly, looking at cranial index we find little if any difference between northern Indian males (70.7–72.3) and southern Indian males (71.3–73.0) or between Indo-Aryan males (70.7–72.2) and Dravidian males (71.3–73.0).

There are, however, some differences between northern and southern Indians in their craniometrics, comparing males with males and females with females, to a degree that is statistically significant, generally speaking (weighted Simes test). Average supraorbital projection (SOS) is larger for northern Indians than southern Indians (Table 7 and Supplementary Table S8). The orbits are on average narrower amongst northern Indians than southern Indians (Table 7 and Supplementary Table S9), whether expressed in terms of their smaller orbital breadth (OBB) or higher orbital index (OBB:OBH). On the other side of the ledger, northern Indian interorbital breadth (DKB) tends to be larger than southern Indian interorbital breadth (Table 7 and Supplementary Table S10). As for facial flatness, northern Indians' dacryon subtense (DKS) is on average smaller than southern Indians', whereas their zygomaxillary subtense (SSS) is relatively large (Table 7 and Supplementary Table S11).

In the preceding comparisons, statistically significant differences between northern and southern Indians are clearer for male than female comparisons. This point applies with even greater force to the two identifiable north-south differences in vault metrics. Male Hindis and Haryanavis have a lower vault length-height index than all southern Indians males, but this difference is less clear when females are compared (Table 7 and Supplementary Table S1). (The average vault length-height index of Punjabi males is also lower than that of any southern Indian male series, but the difference is statistically significant only in comparison with Konkani males; and with the females, Punjabis actually have a significantly higher index than the Malayalam.) Also, northern Indian male frontals tend to be narrower than southern Indian male frontals, as shown by the smaller bistephanic breadth (STB) and maximum frontal breadth (XFB) of northern Indian males (Table 7 and Supplementary Table S3). However, the only reflection of this difference in the female comparisons is female Hindis' significantly smaller XFB compared with the XFB of Telugu, Kannada and Tamil females.

### 3.4. Male Indian Averages Compared to Other Series

To place the craniometric differences between northern and southern Indians in context, this section compares the averages for male Indians with the averages recorded for other series, notably by Howells [17] but also by Warusawithana-Kutilake [7] for Veddas, supplemented by Veddas' simotic index from Woo and Morant [5].

**Figure 3:**
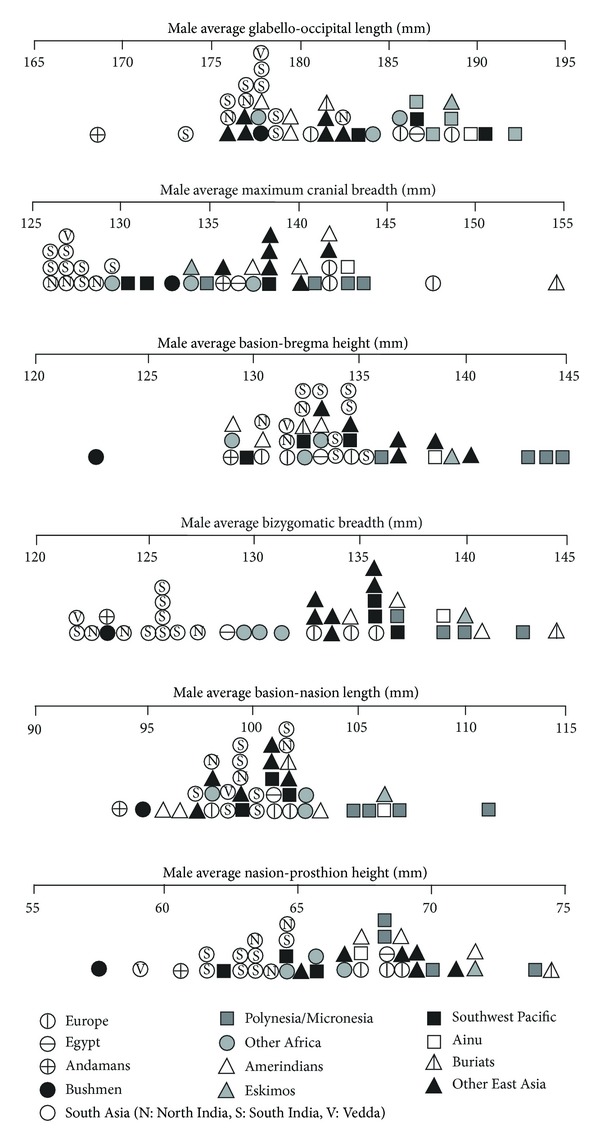
Male South Asians compared to Howells series on some main cranial measurements.

Figure 3 focuses on six main cranial measurements in showing that Indian crania are small by general standards. Indians' breadth measurements are amongst the smallest in the world, and their length measurements and nasion-prosthion height are below average, although their basion-bregma height is moderate. The small size of Indian crania can be related to Indians' generally small body size [34]. Two pygmy populations, the Andamanese and Kalahari Bushmen, have crania that are usually smaller than Indians' except on breadth measurements. The Veddas, who are also small-bodied, have average cranial measurements that either fall within the Indian range or, in the case of nasion-prosthion height, below it.

**Figure 4:**
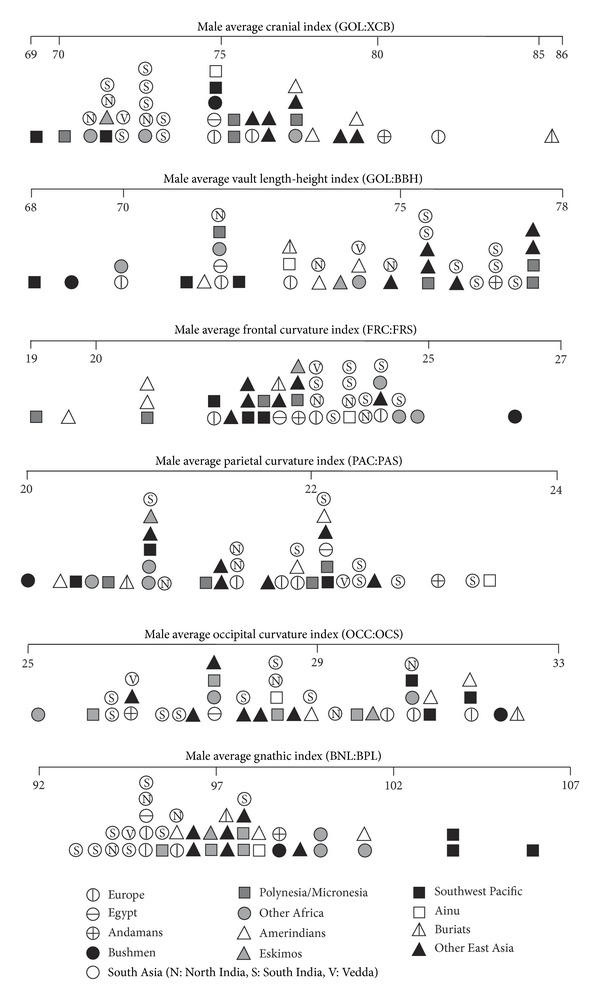
Male South Asians compared to Howells series on indices of the cranial vault.

Figure 4 compares Indians with other series on six indices that have cranial chords as the denominator. Indian cranial vaults are on average narrow (dolichocranic) but relatively tall, at least in the case of southern Indians, and have well-developed frontal curvature but variable parietal and occipital curvature. Indian crania also tend to be orthognathic, with basion-nasion length greater than basion-prosthion length. Veddas consistently fall within the Indian range, but no other series shows the suite of features displayed by Indians. For instance, Southwest Pacific (“Australoid”) series resemble Indians in their narrow vault but differ in their generally lower vault, prognathism, more receding frontal, and more bulging occipital bone.

**Figure 5:**
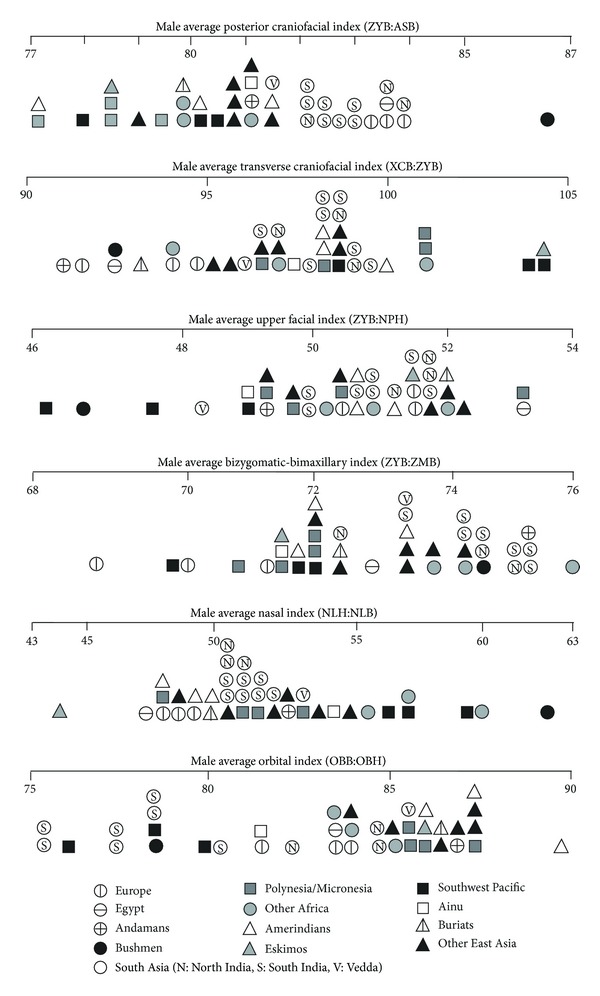
Male South Asians compared to Howells series on indices involving facial chords.

Figure 5 compares Indians with other series on six indices that involve facial chords. Indian crania on average have a moderately wide biasterionic breadth in relation to bizygomatic breadth. Stock et al. [9] also found this to be a feature of Indian crania, but in their analysis it was a feature otherwise shared with Andamanese and Veddas, whereas here we instead find a European/Egyptian (“Caucasoid”) similarity for Indians. Indian crania also have a moderate transverse craniofacial index, narrow face, wide bimaxillary breadth compared to bizygomatic breadth, quite narrow nasal aperture, and variable orbit shape. Indians' variable orbit shape reflects the difference between southern Indians with broad orbits and northern Indians with narrower orbits, noted above. None of the comparative series show the suite of features displayed by Indians. For instance, Caucasoids resemble Indians in their moderately wide biasterionic breadth and narrow faces, but differ in their lower transverse craniofacial index and narrower bimaxillary breadth and nasal aperture.

**Figure 6:**
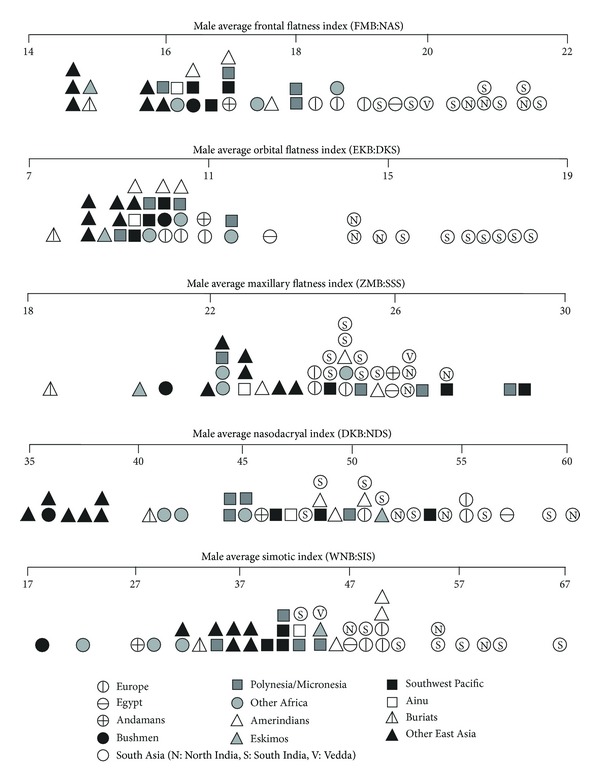
Male South Asians compared to Howells series on indices of facial flatness.

Figure 6 compares Indians with other series on their facial flatness indices. A low index reflects a flat face, as shown particularly by the Buriats, other East Asians, and Kalahari Bushmen, whereas a high index reflects a medially protrusive face. Indians are shown to have faces that are medially very protrusive across the frontal, orbital, and nasal regions and moderately protrusive across the maxilla (lower face). Veddas fall within the Indian range on the three available comparisons. Comparable results were obtained by Woo and Morant [5] and Hanihara [8], who found that South Asians tend to have the highest frontal flatness index in the world, high simotic index, and moderate (zygo/pre) maxillary index. The larger dacryon subtense of southern Indians compared to northern Indians, noted above, is underlined by southern Indians' particularly high orbital flatness index.

Table 8 summarises the index comparisons in Figures 4 to 6. When Indians are compared to other groups represented by more than one series, a similarity is recognised when Indians either fall within the range of the other group or else encompass the range of the other group. Dissimilarity is recognised when the ranges are mutually exclusive. However, when Indians are compared to single series, such as Andamanese or Veddas, similarity is recognised when that series falls near the centre of the Indian range, and dissimilarity is recognised when it falls well away from the Indian range.

Except for Buriats, every group or single series is similar to Indians on at least one index. Europe, Veddas, and Egypt register the largest number of similarities (resp., seven, six, and five). Veddas are dissimilar from Indians on just one index, but every other group or single series is dissimilar on between four and six (Ainu, Egypt, Europe) to 13 indices (Kalahari Bushmen). Veddas would appear to be the non-Indian series most similar to Indians, followed by Caucasoids.

### 3.5. Principal Components Analysis (PCA)

PCA provides a multivariate perspective on the univariate and bivariate comparisons detailed above. The comparisons include all of the better sampled series measured by Howells [17] but exclude the Veddas, for whom we lack access to the original measurements. The six Indian series with the largest sample size—three from northern Indian and three from southern India—are included for analysis. As noted above, northern Indian cranial series are very similar to each other, as are southern Indian cranial series. Therefore, including the less well sampled Indian series would just add noise to the analysis. Further, males and females are analysed separately in view of their shape distinctions, as described previously. In addition, cranial chord fractions are excluded in view of their unreliability as reflected by their excessive variability.

Application of PCA produces very similar results for both males and females. The first component (PC1) accounts for 30% of variability (approximately), the second and third components (PC2 and PC3) for 8% each, and the fourth and fifth components for 5% each (Table 9), with decreasingly smaller amounts for the remaining components. As is common when PCA is applied to biological data [21], PC1 is a size component, with positive weightings on most variables (Table 10). In the present analysis, however, the PC1 weightings for the upper and middle facial subtenses are either negative (NAS, DKS, and SIS) or weakly positive (NDS). That is, large overall cranial size tends to be associated with upper and middle facial flatness. These subtenses also have the highest positive loadings on PC2, followed by cranial lengths, while minimum cranial breadth (WCB), maximum cranial breadth (XCB), and malar subtense (MLS) have the strongest negative loadings. In the case of PC3, which is the second most important of the shape components, cranial breadths and especially frontal breadths have the highest positive loadings (STB, XFB, XCB and AUB), while basion-prosthion length (BPL) has a strong negative loading (Table 10).

**Figure 7:**
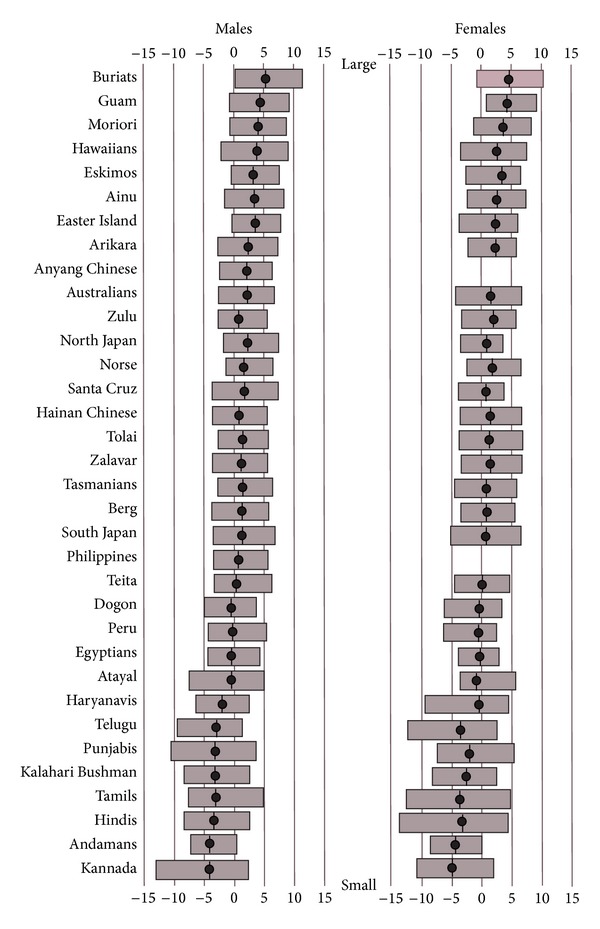
Averages and ranges on PC1 for the Howells and Indian series ordered according to approximate cranial size.

Figure 7 illustrates how the different series score on PC1, arranged in approximate order from the series with the largest crania (Buriats, Guam, Polynesians, Eskimos, and the Ainu of Japan) to the series with the smallest crania (Indians, Kalahari Bushmen and Andamanese). Figure 7 presents the range of PC1 scores for each series as well as the series averages. Inspection of the ranges shows that the smallest specimens in the series with the largest crania are of approximately the same size as the largest specimens in the series with the smallest crania. In the case of the series with intermediate-sized crania, their ranges overlap extensively with the ranges of both the series with large and small crania. Also of interest, there is a difference of around 10 between the series with the largest and the smallest crania in terms of their average PC1 score (Buriats scoring just over 5 compared with Kannada scoring just over −5), and this is similar to the difference of around 10 between the largest and smallest crania within any series (for instance, Buriats ranging between about 10 and 0, and Kannada ranging very approximately between 0 and −10). These observations apply equally to males and females.

**Figure 8:**
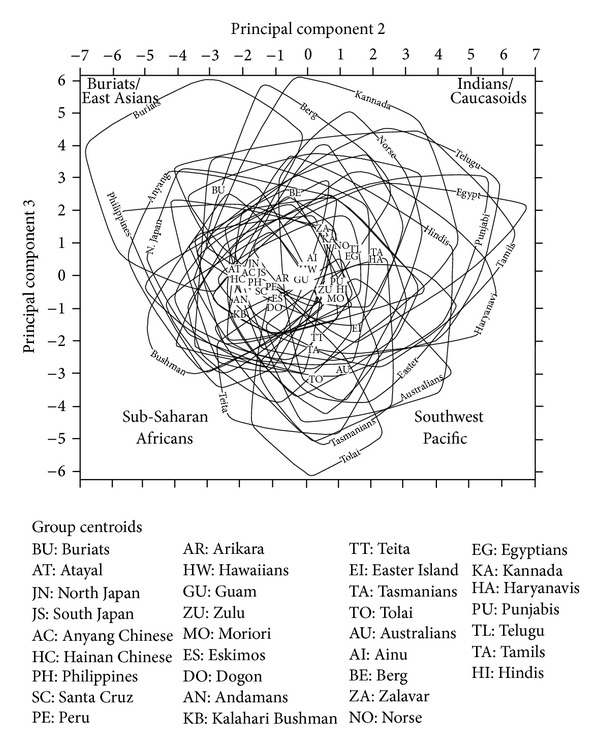
Male centroids and ranges for PC2 and PC3 for the Howells and Indian cranial series.

**Figure 9:**
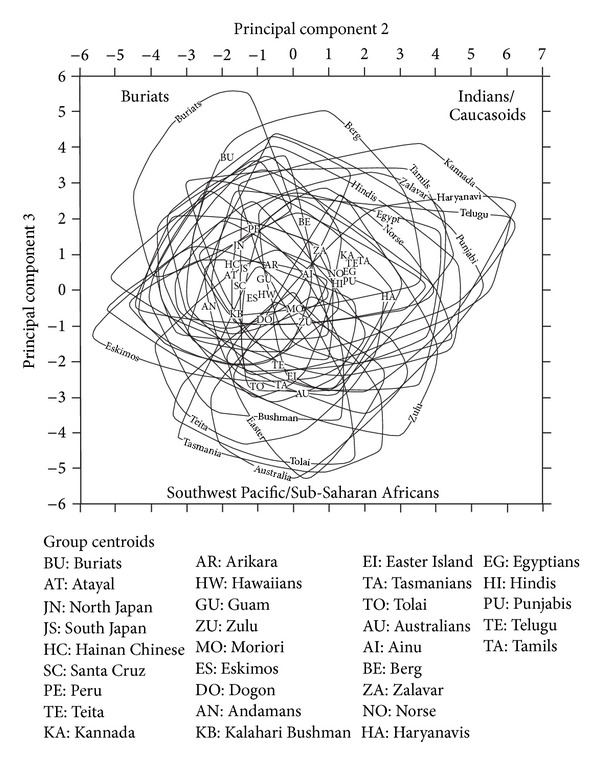
Female centroids and ranges for PC2 and PC3 for the Howells and Indian cranial series.

Figures 8 and 9 illustrate how the series compare the two main shape components, PC2 and PC3. The series are represented both by their centroids and their ranges of variation. For most series, these ranges overlap extensively and so it would be very difficult, and arguably unnecessary, to distinguish them from each other. The only ranges that can be individually labelled are those that relate to series that fall towards the extremes of modern human craniometric variation. Both the centroids and the ranges carry the same message for understanding differences between series in cranial shape. For instance, Buriats are distinguished by a strongly negative score on PC2 and a strongly positive score on PC3. This is shown by the position of the Buriat centroid and also by the Buriat range, with approximately half of the Buriat range of variation falling outside the range of variation documented for any of the other series. Also, while most series overlap with Buriats on their range, some do not, notably Southwest Pacific groups (Australians, Tasmanians, and Tolai), Easter Islanders, and (in the female analysis) the Teita and Zulu from Sub-Saharan Africa.

In accord with the index analysis described above, Indians and Caucasoids align on the two main shape indices. Their centroids are neutral to weakly positive on both PC2 and PC3, and their ranges include the only analysed crania that are strongly positive on both of these PCs (Figures 8 and 9). Their centroids are distant from the Southwest Pacific centroids and, to a lesser degree, the Teita and Easter Island centroids, which are strongly negative on PC3. The Indian and Caucasoid centroids are also quite distant from the Andamanese and Bushman centroids, which are weakly negative on both PC2 and PC3. However, a point of interest is that the Ainu centroid is close to the Indian and Caucasoid centroids on both the male and female analyses.

The graphical representation of the PC2 and PC3 results is of value in reiterating certain observations that emerged from the index analysis, notably the general similarity between Indians and Caucasoids, especially in sharing a medially protrusive face. It is also of value in indicating a central range of human craniometric shape variation, where most of the series comprehensively overlap with each other and where most of the centroids lie. It is however limited in its value in representing only some 16% of craniometric variability. To obtain a more complete picture of the circa 70% of human craniometric variability that is shape rather than size related, we turn to Mahalanobis-*D* distance comparisons of Mosimann indices.

### 3.6. Mahalanobis-*D* Distance Comparisons of Mosimann Indices

Mosimann indices control for size by dividing a specimen's measurements by the geometric mean of all of its measurements [22]. Supraorbital and glabella projection need to be excluded from analysis, as they can measure zero on crania from India (and elsewhere), which prevents calculation of the measurements' geometric mean. Once the measurements were transformed into Mosimann indices, the Mahalanobis *D* ^2^ distances between series were calculated. These distances were then converted into Euclidean distances by calculating their square roots (Mahalanobis-*D* distances). The series were then clustered using average-linkage hierarchical clustering.

Both the male and female dendrograms were seriated, according to a procedure described elsewhere [35]. This procedure involves placing the series and/or clusters most different from each other at opposite poles of the seriation, and, to the extent permitted by the structure of the dendrogram, ordering the other series and/or clusters according to the degree to which they approach one or the other pole. In addition to emphasising extreme differences, the seriation exercise allows for the representation of secondary affinities that may not be captured by the clusters themselves. The success of the seriated order in producing a perfect seriation is measured by the coefficient of variation between the ordered matrix of interseries distances and the closest matrix that could be found with all of the distances perfectly seriated. (The results obtained here, which fall between 70 and 75%, can be described as “fair.”)

Based on the preceding analyses, the following predictions can be made of how Indians should compare with the series measured by Howells. Indians should form a distinct cluster, albeit with northern Indians distinguishable from southern Indians. Indians should be approached by Caucasoids, whereas Buriats and Bushmen should be far removed.

**Figure 10:**
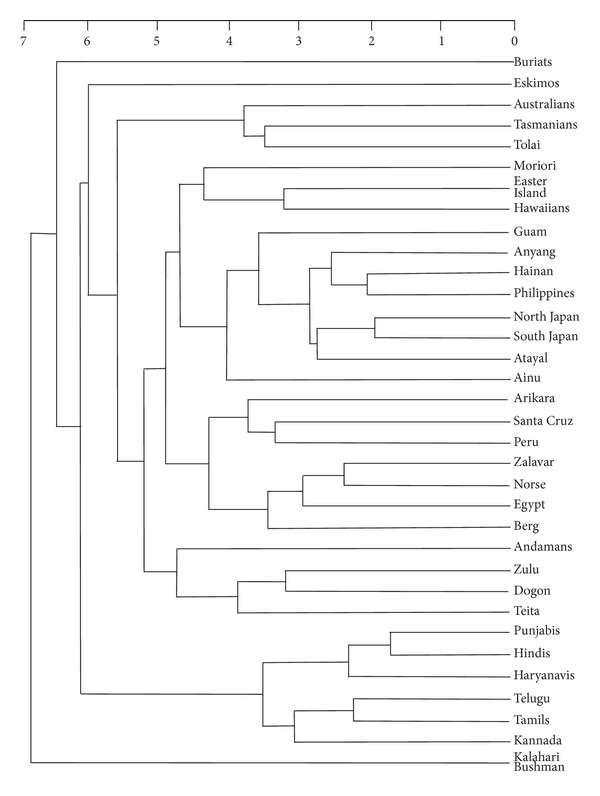
Seriated average-linkage hierarchical dendrogram, Mahalanobis-*D* distances for 34 male series, Mosimann indices. (Coefficient of variation with a perfect seriation 72.3%.)

**Figure 11:**
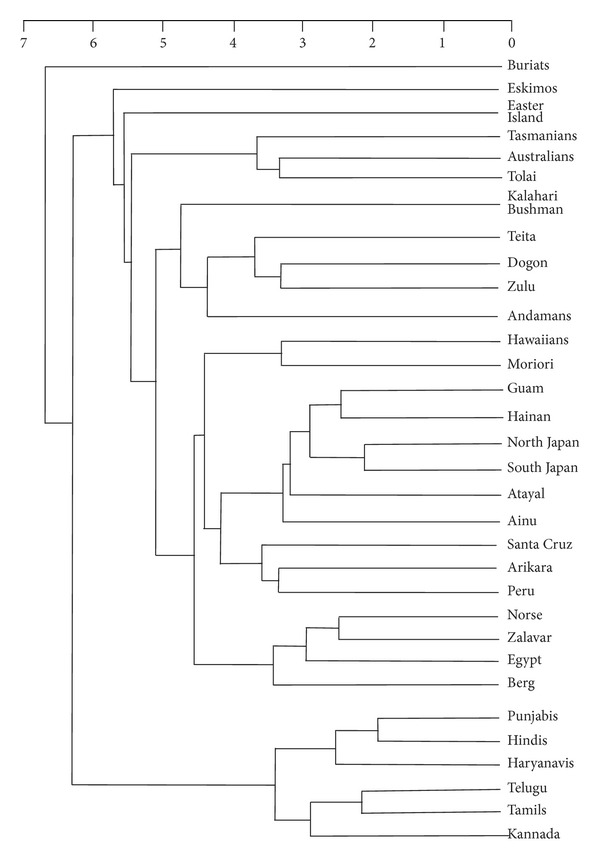
Seriated average-linkage hierarchical dendrogram, Mahalanobis-*D* distances for 32 female series, Mosimann indices. (Coefficient of variation with a perfect seriation 74.6%.)

The seriated dendrograms (Figures 10 and 11) agree in certain fundamental respects. The three northern Indian series cluster together, as do the three southern Indian clusters, before joining together into a distinct Indian cluster. Also, southern Indians lie at one pole, whereas northern Indians are intermediate between southern Indians and other series (see below). In addition, Egyptians join the three European series to make a Caucasoid cluster, while the Andamanese join the Dogon, Teita, and Zulu of Sub-Saharan Africa to form a distinct cluster. Another similarity between the male and female analyses is that Buriats, Eskimos, Easter Islanders, and Southwest Pacific series (Australians, Tasmanians, and Tolai) are the series most distant from Indians.

Where Figures 10 and 11 disagree is in the secondary affinity suggested for Indians. In the male analysis, Indians are flanked by Sub-Saharan African series (here counting Bushmen), whereas in the female analysis, Indians plot adjacently to Caucasoids. Inspection of the original distances (Tables 11 and 12) shows that the female analysis is the more informative from the perspective of revealing Indians' wider affinities. Both male and female Indians are closer to Europeans and Egyptians than to any other analysed series, notably Bushmen. The reason why this fact does not emerge from the male analysis (Figure 10) is because the craniometric distances between Indians and Sub-Saharan Africans are smaller for males than females (Tables 11 and 12). Accordingly, the male seriated dendrogram emphasises the craniometric distance between Buriats/Eskimos and Sub-Saharan Africans, overriding the craniometric difference between Indians and Sub-Saharan Africans, whereas in the female analysis these emphases are reversed.

The similarity between Indian and Andamanese crania proposed by previous multivariate studies [6, 9, 10] cannot be confirmed by our analysis. Instead, Andamanese cluster with Sub-Saharan Africans, as originally observed by Howells [3], whereas Indians are more similar to Caucasoids than to any other populations outside of South Asia. The reason for the difference between our findings and those of previous studies on Indian craniometrics may be the larger number of variables in our analysis presented here, 42 compared with 20 to 30. Further, there is no evidence for the southern Indian-Andamanese affinity that would have been expected from the genetic comparisons of Reich et al. [16]. With barely an exception, southern Indians register a greater craniometric distance from Andamanese than northern Indians do, just as southern Indians are more distant than northern Indians from any other population outside of South Asia (Tables 11 and 12).

## 4. Discussion

The literature review in our Introduction generated three hypotheses for our craniometric analysis. The first hypothesis, the distinction between northern and southern Indians within a discrete Indian cluster, was unequivocally confirmed. The second hypothesis was confirmed as a secondary “Caucasoid” affinity emerged for northern Indians. However, the expectation from the third hypothesis of a secondary Andamanese affinity for southern Indians was falsified.

The craniometric differences found here between northern and southern Indians are not in terms that might have been expected from the comparative literature. For instance, based on Bharati et al. [36], narrower crania might have been expected in the south than the north, but instead all of the sampled Indian series were found to have similarly narrow crania. The explanation for this result may be that all of these Indian populations inhabit hot climates [37], even if the torrid heat of northern India's lowlands is a seasonal phenomenon. If there is an adaptive basis for the differences that southern Indians show from northern Indians, such as broader orbits that are medially more protrusive, this basis remains to be explored.

The distinctiveness of Andamanese and southern Indian crania need not challenge the finding by Reich et al. [16] for an “Ancestral South Indian” ancestry shared by southern Indians and Andamanese. The point is that some populations are craniometrically specialised while others are not. The specialised nature of Buriat craniometrics, which is very clear both from index and multivariate analysis (Figures 4 to 11), has been noted previously [6]. What the present analysis adds is that southern Indians also have specialised craniometrics. Andamanese on the other hand have unspecialised craniometrics, as shown by how they cluster with geographically distant Sub-Saharan Africans, and seriate adjacently to the central bloc of recent human crania (consisting of Caucasoids, Amerindians, and populations from Japan and China to Taiwan and parts of the Pacific). Therefore, southern Indians' craniometric distinctiveness from Andamanese should be interpreted as a result of their craniometric specialisation rather than as evidence against a shared, ancient ancestry with Andamanese.

Populations with medially protrusive upper and middle faces are distributed from Scandinavia to the circum-Mediterranean, India, and Sri Lanka [5, 8]. This indicates the existence of a population complex extending from Scandinavia south-south-east to Sri Lanka. Gene flow across this continuously populated region would have been relatively uninterrupted in comparison to the formidable barriers to gene flow presented by the Atlantic Ocean to the northwest, the Sahara Desert to the southwest, and the Himalayas and Eurasian Steppe to the northeast. Upper and middle facial protrusion are developed particularly strongly in southern India (Figure 6). This observation is not explicable in terms of a contribution to the southern Indian gene pool from Central Asia and/or the Mediterranean. On the other hand, the intermediate position of northern Indians between southern Indians, and Caucasoids northwest of India, could be explicable in terms of the incursion of Indo-European (Indo-Aryan) speakers into northern India from the northwest, or alternatively it could simply reflect clinal variation.

If there were an Australoid “substratum” component to Indians' ancestry, we would expect some degree of craniometric similarity between Howells' Southwest Pacific series and Indians. But in fact, the Southwest Pacific and Indian are craniometrically very distinct, falsifying any claim for an Australoid substratum in India. Only the “Veddoid” substratum component invoked by some theories would be potentially supported, based on the index similarities between Veddas and Indians. The problem with this proposal is that, craniometrically, the Veddas should be viewed as just another South Asian population. The basis for invoking the Veddas as representative of a substratum component, rather than the Kannada and Tamils (for instance), appears difficult to justify. Noteworthy in this context is the accumulating evidence that the Dravidian languages have a preagricultural origin in southern India and dispersed with the expansion of the southern Indian Neolithic [38].

We recognise that craniometric data are not as powerful as genetic data in unmasking populations' biological relatedness. Indeed, where our results could not duplicate the affinity between southern Indians and Andaman Islanders suggested by genetic data, we attributed the discrepancy to southern Indians' craniometric specialisation. However, craniometric data have particular value for the analysis of ancient South Asian burials [13] which may not support genetic analysis if none of their ancient DNA has survived. As documented here, South Asian crania are characterised by a distinctive suite of features which include tall, narrow vaults, and medially protrusive upper and middle faces. The hypothesis of predominantly local genetic origins for South Asian populations predicts that these features will also characterise prehistoric burials. This point provides direction on where to focus attention in future studies on India's prehistoric burials. At the same time, we should be mindful of how variable crania within any Indian series can be in their shape, an aspect that presumably also applied in the past. Therefore, when analysing a single specimen, we should be duly cautious in how much weight to place on the outcome, and when analysing a series we should expect some healthy variability in the results.

## 5. Conclusions

Craniometric variability within any Indian series is considerable, but between Indian series it is slight for most measurement and indices. Craniometric differences within India boil down to a primary distinction between northern and southern Indians. When crania from outside India are considered, the Veddas are similar enough to be grouped with Indians as “South Asians.” Otherwise, Caucasoid series from Egypt and Europe are closest to Indians, especially northern Indians. The similarity between these Caucasoids and northern Indians would be expected from geographical considerations, but it may also reflect some degree of population incursion into northern India associated with the introduction of Indo-Aryan languages. Southern Indians have specialised craniometrics otherwise revealed only by other South Asians. Craniometric analysis thus accords with recent genetic studies that point to a predominantly indigenous component in Indians' biological ancestry.

## Supplementary Material

The supplementary material comprises 13 tables with sample sizes, means, standard deviations and ranges for male and female Punjabis, Haryanavis, Hindis, Urdu, Konkanis, Telugu, Kannada, Tulu, Tamils and Malayalam. These basic statistical data are provided for the Howells measurements and indices listed in Table 2 of the main text. There are five tables (Tables S1 to S5) focused on measurements and indices of the cranial vault, five tables (Tables S7 and S9 to S12) focused on measurements and indices of the facial skeleton, and three tables (Tables S6, S8 and S13) that combine vault and facial measurements and/or indices.

## Figures and Tables

**Table 1:** Indian series included in the present study.

Series	Location	Language group	Holding institutions
Punjabi	Northwest India	Indo-Aryan	Panjab: Government Medical College, Patiala; Christian Medical College, Ludhiana. Chandigarh: Government Medical College. New Delhi: Mulana Azad Medical College.
Haryanavi	Northwest India	Indo-Aryan	Haryana: Post Graduate Institute of Medical Sciences, Rohtak.
Hindi	North India	Indo-Aryan	New Delhi: Maulana Azad Medical College, Lady Harding Medical College. Uttar Pradesh: King George Medical College, Lucknow; University of Allahabad, Allahabad; Moti Lal Nehru Medical College, Allahabad. Madhya Pradesh: Gandhi Medical College, Bhopal.
Urdu	South India	Indo-Aryan	Andhra Pradesh: Osmania Medical College, Hyderabad; Gandhi Medical College, Secunderabad.
Konkani	South India	Indo-Aryan	Karnataka: Kasturba Medical College, Manipal; Kasturba Medical College, Mangalore.
Telugu	South India	Dravidian	Andhra Pradesh: Osmania Medical College, Hyderabad; Gandhi Medical College, Secunderabad; Sri Venkateshwara University, Tirupathi; Siddarth Medical College, Vijayawada, Andhra Pradesh; NRI Medical College, Chinakakani; DR. PSIMS & Rf, Krishna. Adelaide: South Australian Museum.
Kannada	South India	Dravidian	Karnataka: St John's Medical College, Bangalore; Kasturba Medical College, Mangalore.
Tulu	South India	Dravidian	Karnataka: Kasturba Medical College, Manipal; Kasturba Medical College, Mangalore.
Tamil	South India	Dravidian	Pondicherry: Jawaharlal Nehru Institute of Medical Education and Research. Tamil Nadu: Madras Medical College, Chennai; Christian Medical College, Vellore. Adelaide: South Australian Museum.
Malayalam	South India	Dravidian	Kerala: Government Medical College, Calicut.

**Table 2:** Measurements and indices included in this study.

Measurement/index	Acronym
Maximum glabello-occipital cranial length	GOL
Maximum nasio-occipital cranial length	NOL
Basion-nasion (cranial base) length	BNL
Basion-bregma cranial height	BBH
Maximum transverse cranial breadth (above the supramastoid crests)	XCB
Maximum transverse frontal breadth	XFB
Bistephanic breadth (frontal breadth across the inferior temporal lines)	STB
Bizygomatic facial breadth	ZYB
Biauricular breadth (across the roots of the zygomatic processes)	AUB
Minimum cranial breadth (across the infratemporal crests)	WCB
Biasterionic (maximum occipital) breadth	ASB
Basion-prosthion (facial) length	BPL
Nasion-prosthion (upper facial) height	NPH
Nasal height	NLH
Orbital height (left)	OBH
Orbital breadth from dacryon (left)	OBB
Bijugal breadth (breadth across the middle malars)	JUB
Nasal breadth	NLB
External palate breadth	MAB
Mastoid process height	MDH
Mastoid process breadth	MDB
Bimaxillary (inferior malar) breadth	ZMB
Zygomaxillary subtense (subspinale projection from bimaxillary breadth)	SSS
Bifrontal (upper facial) breadth	FMB
Nasion-frontal subtense (nasion projection from binfrontal breadth)	NAS
Biorbital breadth (breadth from dacryon to ectoconchion)	EKB
Dacryon subtense (dacryon projection from biorbital breadth)	DKS
Interorbital breadth (across the dacrya)	DKB
Nasodacryal subtense (least projection of nasal bones from interorbital breadth)	NDS
Simotic chord (least breadth across the nasal bones)	WNB
Simotic subtense (projection of the nasal bridge from simotic chord)	SIS
Inferior malar length (left)	IML
Maximum malar length (left)	XML
Malar subtense (greatest projection of malar from maximum malar length)	MLS
Cheek height (left)	WMH
Supraorbital projection (projection of left superciliary ridge)	SOS
Glabella projection (greatest projection from nasion-supraglabellare chord)	GLS
Foramen magnum (basion to opisthion) length	FOL
Frontal (nasion to bregma) chord	FRC
Frontal subtense (greatest projection from frontal chord)	FRS
Frontal fraction (distance from nasion where greatest frontal projection falls)	FRF
Parietal (bregma to lambda) chord	PAC
Parietal subtense (greatest projection from parietal chord)	PAS
Parietal fraction (distance from bregma where greatest parietal projection falls)	PAF
Occipital (lambda to opisthion) chord	OCC
Occipital subtense (greatest projection from occipital chord)	OCS
Occipital fraction (distance from lambda where greatest occipital projection falls)	OCF
Cranial index (100 ∗ XCB/GOL)	GOL:XCB
Vault length-height index (100 ∗ BBH/GOL)	GOL:BBH
Frontal curvature index (100 ∗ FRS/FRC)	FRC:FRS
Parietal curvature index (100 ∗ PAS/PAC)	PAC:PAS
Occipital curvature index (100 ∗ OCS/OCC)	OCC:OCS
Gnathic index (100 ∗ BPL/BNL)	BNL:BPL
Posterior craniofacial index (100 ∗ ASB/ZYB)	ZYB:ASB
Transverse craniofacial index (100 ∗ ZYB/XCB)	XCB:ZYB
Upper facial index (100 ∗ NPH/ZYB)	ZYB:NPH
Bizygomatic-bimaxillary index (100 ∗ ZMB/ZYB)	ZYB:ZMB
Nasal index (100 ∗ NLB/NLH)	NLH:NLB
Orbital index (100 ∗ OBH/OBB)	OBB:OBH
Frontal flatness index (100∗ NAS/FMB)	FMB:NAS
Orbital flatness index (100∗ DKS/EKB)	EKB:DKS
Maxillary flatness index (100∗ SSS/ZMB)	ZMB:SSS
Nasodacryal index (100∗ NDS/DKB)	DKB:NDS
Simotic index (100∗ SIS/WNB)	WNB:SIS

**Table 3:** Relationship between measurements' mean and standard deviation for Indian series.

Measurements included	Untransformed variables		Variables transformed to base 10 logarithms	
	Pearson's *r*	Slope of best-fit line	Pearson's *r*	Slope of best-fit line
All measurements, all series	0.743	0.033	0.839	0.429
All measurements except fractions, all series	0.812	0.034	0.861	0.424
All measurements except fractions, all series with ≥30 values per measurement	0.887	0.032	0.907^a^	0.411^b^

^a^Pearson's *r* value is 0.908 for northern Indians and 0.907 for southern Indians.

^b^Slope of best-fit line is 0.408 for northern Indians and 0.413 for southern Indians.

**Table 4:** Relationship between measurements' mean and range for Indian series.

Linear measurements included	Untransformed variables		Variables transformed to base 10 logarithms	
	Pearson's *r*	Slope of best fit line	Pearson's *r*	Slope of best fit line
All measurements, all series	0.699	0.142	0.752	0.438
All measurements except fractions, all series	0.752	0.146	0.767	0.433
All measurements except fractions, all series with ≥30 values per measurement	0.852	0.166	0.878^a^	0.420^b^

^a^Pearson's *r* value is 0.883 for northern Indians and 0.876 for southern Indians.

^b^Slope of best fit line is 0.431 for northern Indians and 0.411 for southern Indians.

**Table 5:** Relationship between indices' mean and variability (other than nasal flatness indices) for Indian series.

Series included and measure of variability compared	Untransformed variables		Variables transformed to base 10 logarithms	
	Pearson's *r*	Slope of best fit line	Pearson's *r*	Slope of best fit line
All series (standard deviations)	0.644	0.028	0.684	0.369
All series with ≥30 values per index (standard deviations)	0.740	0.028	0.781^a^	0.367^b^
All series (ranges)	0.509	0.116	0.493	0.351
All series with ≥30 values per index (ranges)	0.637	0.126	0.684^c^	0.327^d^

^a^Pearson's *r* value is 0.797 for northern Indians and 0.768 for southern Indians.

^b^Slope of best fit line is 0.371 for northern Indians and 0.364 for southern Indians.

^c^Pearson's *r* value is 0.725 for northern Indians and 0.660 for southern Indians.

^d^Slope of best fit line is 0.345 for northern Indians and 0.316 for southern Indians.

**Table 6:** Count of measurements for which males are statistically significantly larger than females in Indian series.

	Sexual dimorphism					
Series	Universal^a^	Universal if well sampled^b^	Typical if well sampled^c^	Weak^d^	Unclear^e^	Total for 47 measurements
Konkani	6	2	5	0	0	13/47 (27.7%)
Urdu	6	2	3	0	0	11/47 (23.4%)
Malayalam	6	7	1	0	0	14/47 (29.8%)
Tulu	6	16	9	1	0	32/47 (68.1%)
Telugu	6	16	12	3	0	37/47 (78.7%)
Haryanavi	6	16	5	1	0	28/47 (59.6%)
Punjabi	6	16	7	2	0	31/47 (66.0%)
Kannada	6	16	10	3	0	35/47 (74.5%)
Tamil	6	16	11	3	0	36/47 (76.6%)
Hindi	6	16	12	5	0	39/47 (83.0%)

^a^BBH, AUB, MDH, ZYB, JUB, and OBB (males significantly larger in every series).

^b^GOL, XCB, NOL, ASB, OCF, MDB, BNL, NPH, FMB, GLS, EKB, NLH, NLB, WCB, XML, and WMH (males significantly larger in every series with at least 30 males).

^c^PAC, STB, XFB, FRC, FRF, BPL, SOS, ZMB, NDS, SIS, MLS, MAB, and IML (males significantly larger in 5 to 6 series with at least 30 males).

^d^PAS, PAF, OCC, OCS, NAS, DKB, DKS, and SSS (males significantly larger in just 1 to 4 series with at least 30 males).

^e^FRS, FOL, OBH, and WNB (males not significantly larger in any series).

**Table 7:** Significant craniometric differences between northern and southern Indians.

Measurement or index	Males (in bold if northern Indians values higher)	Females (in bold if northern Indians values higher)
SOS	**All comparisons except Haryanavis cf. Konkanis and Telugu**	**All comparisons except Hindis and Haryanavis cf. Konkanis and Urdu**
OBB	All comparisons	Punjabis cf. Telugu; Haryanavis cf. Telugu and Malayalam; Hindis cf. Konkanis and Telugu
OBB:OBH	**All comparisons**	**All comparisons involving Telugu, Tulu, Tamils and Malayalam, plus Punjabis and Haryanavis cf. Kannada **
DKB	**All comparisons involving Telugu, Konkanis, Tamils, and Malayalam**	**All comparisons involving Telugu, Tulu and Tamils, plus Punjabis and Haryanavis cf. Kannada, and Haryanavis cf. Malayalam**
DKS	All comparisons except Haryanavis cf. Telugu and Malayalam	All comparisons involving Hindis, plus Haryanavis cf. Malayalam
SSS	**All comparisons involving Tulu and Kannada, plus Haryanavis cf. Tamils and Malayalam**	**All comparisons involving Haryanavis, plus Punjabis cf. Tulu and Malayalam, and Hindis cf. Tulu**
BBH:GOL	All comparisons involving Hindis and Haryanavis, plus Punjabis cf. Konkanis	Punjabis cf. Kannada and **Malayalam**; Haryanavis cf. Konkanis, Kannada, Tulu and Tamils; Hindis cf. Konkanis, Kannada and Tamils
STB	All comparisons involving Punjabis and Hindis, plus Haryanavis cf. Tulu	No comparisons
XFB	All comparisons involving Hindis, plus Punjabis and Haryanavis cf. Kannada, Tulu and Tamils, and Punjabis cf. Konkanis and Telugu	Hindis cf. Telugu, Kannada and Tamils

**Table 8:** Comparisons between Indians and Worldwide Series on Average Indices (Males)^a^.

Index	Indians' variability	Similarities	Dissimilarities
Cranial	Narrow	Africa, Eskimos, Southwest Pacific, Veddas	Amerindians, Andamans, Buriats, East Asia, and Europe
Vault length-height	Wide	Polynesia/Micronesia, Veddas	Bushmen
Frontal curvature	Narrow	Ainu	Amerindians, Bushmen, Polynesia/Micronesia, and Southwest Pacific
Parietal curvature	Wide	Egypt, Europe, Veddas	Bushmen
Occipital curvature	Wide	Ainu, East Asia	Buriats, Bushmen
Gnathic	Moderate	Egypt, Europe, Polynesia/Micronesia, Veddas	Africa, Southwest Pacific
Posterior craniofacial	Narrow	Europe	Africa, Amerindians, Buriats, Bushmen, Eskimos, Polynesia/Micronesia, and Southwest Pacific
Transverse craniofacial	Narrow	Africa, Ainu	Andamans, Buriats, Bushmen, Egypt, Eskimos, and Europe
Upper facial	Moderate	Amerindians, East Asia, Europe	Bushmen, Egypt, Southwest Pacific, and Veddas
Bizygomatic-bimaxillary	Moderate	Bushmen, Veddas	Ainu, Europe, Southwest Pacific, and Polynesia/Micronesia
Nasal	Narrow	East Asia, Polynesia/Micronesia	Africa, Bushmen, Egypt, Eskimos, Europe, and Southwest Pacific
Orbital	Wide	Ainu, Bushmen, Europe, Southwest Pacific	Amerindians, Andamans, and Polynesia/Micronesia
Frontal flatness	Wide	None	All except Veddas and Egypt
Orbital flatness	Wide	None (Vedda comparison unavailable)	All except Veddas (comparison unavailable)
Maxillary flatness	Moderate	Andamans, Egypt, Veddas	Ainu, Buriats, Bushmen, East Asia, and Eskimos
Nasodacryal	Wide	Amerindians, Egypt, Eskimos, Europe (Vedda comparison unavailable)	Africa, Buriats, Bushmen, and East Asia
Simotic	Wide	Amerindians, Europe	Africa, Andamans, Buriats, Bushmen, East Asia, and Southwest Pacific

^a^“Africa” and “East Asia” in this table respectively correspond to “other Africa” and “other East Asia” in Figures 3–5.

**Table 9:** Variability (per cent) explained by the first five principal components.

Sex	PC1	PC2	PC3	PC4	PC5
Males	30.1	8.0	6.8	5.1	4.8
Females	27.5	8.3	7.7	5.4	5.2

**Table 10:** Factor loadings of the measurements^a^ on the first three principal components.

Measurement	PC 1 Males	PC 2 Males	PC 3 Males	PC 1 Females	PC 2 Females	PC 3 Females
NAS	−0.126	0.766	0.030	−0.079	0.769	−0.212
DKS	−0.391	0.652	0.123	−0.275	0.716	−0.101
SIS	−0.134	0.588	0.218	−0.084	0.642	0.127
NDS	0.092	0.483	0.023	0.171	0.473	−0.071
GOL	0.691	0.485	−0.107	0.650	0.411	−0.259
NOL	0.697	0.468	−0.073	0.677	0.400	−0.214
WNB	−0.020	0.427	0.063	−0.033	0.414	−0.119
SSS	0.126	0.431	−0.189	0.084	0.351	−0.272
BNL	0.627	0.429	−0.148	0.642	0.346	−0.226
FRC	0.509	0.328	0.317	0.493	0.399	0.202
PAC	0.364	0.301	−0.080	0.271	0.313	−0.282
OBB	0.437	0.323	0.061	0.509	0.257	−0.093
FRS	−0.085	0.184	0.366	−0.044	0.345	0.217
BBH	0.523	0.205	0.135	0.462	0.273	0.049
STB	0.232	0.040	0.770	0.294	0.269	0.664
OCS	0.359	0.191	−0.055	0.301	0.050	−0.002
XFB	0.392	−0.031	0.765	0.424	0.235	0.670
NLH	0.659	0.044	0.251	0.671	0.137	0.293
PAS	0.044	0.057	0.023	0.027	0.122	−0.160
BPL	0.626	0.177	−0.473	0.618	−0.043	−0.524
FMB	0.762	0.112	−0.118	0.744	0.072	−0.263
MDH	0.442	0.051	−0.007	0.393	0.084	0.097
OCC	0.493	0.108	0.085	0.426	0.004	0.134
NPH	0.671	0.008	0.199	0.678	0.082	0.236
GLS	0.356	0.085	−0.295	0.302	−0.094	−0.341
EKB	0.818	0.009	−0.140	0.800	−0.042	−0.244
FOL	0.427	−0.019	0.096	0.379	0.084	0.128
OBH	0.469	−0.059	0.219	0.467	0.004	0.269
DKB	0.467	−0.060	−0.184	0.348	−0.030	−0.280
ASB	0.654	−0.066	0.248	0.607	−0.006	0.306
NLB	0.478	−0.032	−0.254	0.449	−0.117	−0.299
IML	0.575	0.005	−0.386	0.545	−0.173	−0.423
MDB	0.569	−0.029	−0.104	0.542	−0.144	−0.030
XML	0.713	−0.030	−0.218	0.681	−0.161	−0.209
MAB	0.705	−0.122	−0.098	0.640	−0.146	−0.059
ZMB	0.697	−0.158	−0.088	0.642	−0.114	−0.026
WMH	0.630	−0.140	0.092	0.609	−0.193	0.218
JUB	0.892	−0.180	−0.086	0.860	−0.153	−0.096
AUB	0.803	−0.191	0.281	0.785	−0.118	0.346
ZYB	0.892	−0.183	0.037	0.869	−0.160	0.099
SOS	0.508	−0.124	−0.304	0.456	−0.219	−0.324
WCB	0.583	−0.287	0.280	0.587	−0.101	0.370
XCB	0.687	−0.307	0.424	0.667	−0.194	0.449
MLS	0.517	−0.296	−0.271	0.472	−0.351	−0.219

^a^Measurements in approximate order (weighted across males and females) from a large positive loading to a large negative loading on the second principal component.

**Table 11:** Mahalanobis-*D* distances after seriation, Mosimann indices, males, Indians, and Kalahari Bushmen compared with each other and with other series.

Series	Punjabis	Hindis	Haryanavis	Telugu	Tamils	Kannada	Bushmen
Buriats	7.054	7.079	7.389	7.791	8.067	8.099	7.154
Eskimos	6.232	6.148	6.717	6.655	7.037	7.340	7.815
Australians	5.615	5.685	5.939	6.433	6.821	7.153	6.603
Tasmanians	6.077	6.169	6.345	6.664	7.070	7.637	6.440
New Britain Tolai	5.758	5.770	6.299	6.320	6.753	7.178	7.162
Moriori	6.411	6.377	6.292	7.240	7.717	7.819	8.128
Easter Island	5.573	5.477	5.460	6.186	6.798	7.019	7.381
Hawaiians	5.563	5.650	5.850	6.210	6.743	6.746	7.363
Guam	5.157	5.207	5.625	5.868	6.511	6.589	7.246
Anyang Chinese	5.886	5.999	6.498	6.723	7.214	7.035	6.748
Hainan Chinese	5.067	5.220	5.832	6.270	6.716	6.537	6.761
Philippines	4.935	5.092	5.718	6.053	6.501	6.466	6.226
North Japan	5.157	5.414	6.059	6.602	7.067	6.906	6.449
South Japan	4.743	4.911	5.566	5.957	6.440	6.349	6.438
Taiwan Atayal	4.912	5.024	5.702	6.083	6.651	6.682	6.134
Ainu	4.547	4.679	5.044	5.401	6.065	6.217	5.912
Arikara (America)	5.438	5.273	5.550	6.272	6.610	6.882	7.799
Santa Cruz (America)	5.166	5.241	5.630	6.500	6.754	7.015	6.906
Peru (America)	4.871	4.802	5.038	6.013	6.371	6.501	7.224
Zalavár (Europe)	3.795	3.648	4.061	4.693	5.349	5.766	6.022
Norse (Europe)	4.425	4.278	4.067	5.317	5.943	6.227	6.210
Egypt	4.214	4.257	4.201	5.125	5.628	5.836	6.619
Berg (Europe)	4.986	4.983	5.049	5.829	6.335	6.619	6.208
Andaman Islanders	5.125	5.801	6.275	6.417	6.993	6.908	6.153
Zulu (Africa)	4.555	4.971	5.494	5.948	6.399	6.421	5.060
Dogon (Africa)	5.745	6.290	6.580	6.901	7.420	7.216	5.670
Teita (Africa)	5.084	5.216	5.381	6.240	6.479	6.421	5.366
Punjabis		1.615	2.624	3.353	3.618	3.392	6.631
Hindis			1.972	2.867	3.074	3.397	7.053
Haryanavis				3.583	3.594	3.819	7.234
Telugu					2.190	3.484	7.520
Tamils						2.430	7.705
Kannada							7.785

**Table 12:** Mahalanobis *D*-distances after seriation, Mossiman indices, females, and Indians compared with each other and with other series.

Series	Hindis	Punjabis	Haryanavis	Telugu	Tamils	Kannada
Buriats	7.768	7.937	8.365	8.071	8.842	8.558
Eskimos	6.452	6.710	7.128	6.650	7.298	7.452
Easter Island	6.477	6.587	6.456	7.204	7.591	7.758
Tasmanians	6.601	6.706	6.836	6.892	7.683	8.139
Australians	5.687	5.959	6.270	6.299	7.163	7.195
New Britain Tolai	6.031	6.330	6.579	6.822	7.497	7.363
Kalahari Bushmen	6.730	6.968	7.607	7.225	7.971	7.968
Teita (Africa)	6.170	6.558	6.765	6.922	7.419	7.231
Dogon (Africa)	6.230	6.410	6.975	6.882	7.530	7.411
Zulu (Africa)	5.244	5.607	6.089	6.258	6.814	6.751
Andaman Islanders	6.572	6.513	7.319	6.833	7.660	7.539
Mokapu Hawaiians	6.471	6.374	6.744	6.718	7.359	7.269
Moriori	6.387	6.329	6.179	6.678	7.502	7.394
Guam	5.632	5.678	6.075	6.181	6.909	6.802
Hainan Chinese	5.697	5.820	6.410	6.360	6.941	6.869
North Japan	5.741	5.634	6.484	6.469	7.301	7.147
South Japan	5.578	5.694	6.341	6.268	7.082	6.981
Taiwan Atayal	5.244	5.268	6.022	5.987	6.686	6.758
Ainu	5.024	5.117	5.759	5.367	6.293	6.432
Santa Cruz (America)	6.135	6.300	6.685	6.785	7.765	7.603
Arikara (America)	5.415	5.732	5.716	5.933	6.809	6.510
Peru (America)	5.656	6.027	6.038	6.394	7.244	6.882
Norse (Europe)	4.625	4.944	4.855	5.177	6.175	6.127
Zalavár (Europe)	4.482	4.785	5.115	5.225	6.192	6.111
Egypt	4.823	5.039	5.217	5.339	6.314	6.211
Berg (Europe)	5.352	5.655	5.796	6.049	6.958	6.826
Hindis		1.887	2.238	2.892	3.196	3.123
Punjabis			2.743	2.725	3.207	3.137
Haryanavis				3.620	3.862	3.681
Telugu					2.099	3.134
Tamils						2.751
