# Supplementary material for: Indian Craniometric Variability and Affinities
Source: Int J Evol Biol. 2013 Dec 24;2013:836738. doi: 10.1155/2013/836738 (PMC3886603; doi:10.1155/2013/836738)
Supplement: Supplementary file 1 — The supplementary material comprises 13 tables with sample sizes, means, standard deviations and ranges for male and female Punjabis, Haryanavis, Hindis, Urdu, Konkanis, Telugu, Kannada, Tulu, Tamils and Malayalam. These basic statistical data are provided for the Howells measurements and indices listed in Table 2 of the main text. There are five tables (Tables S1 to S5) focused on measurements and indices of the cranial vault, five tables (Tables S7 and S9 to S12) focused on measurements and indices of the facial skeleton, and three tables (Tables S6, S8 and S13) that combine vault and facial measurements and/or indices. [file 836738.f1.pdf]

SUPPLEMENTARY MATERIAL: CRANIOMETRIC DATA TABLES FOR INDIAN SERIES

Table S1. Indian series main cranial vault measurements and indices – means, standard deviations and ranges

Series	♂ GOL (mm)	♀ GOL (mm)	♂ XCB (mm)	♀ XCB (mm)	♂ BBH (mm)	♀ BBH (mm)	♂ GOL: XCB	♀ GOL: XCB	♂ GOL: BBH	♀ GOL: BBH
Punjabi ♂ n=100–119, ♀ n=58–71	176.5 ±8.76 150–198	171.2 ±7.30 150–187	127.4 ±6.00 113–141	123.6 ±6.32 103–141	131.6 ±5.77 121–147	128.9 ±4.57 116–141	72.3 ±4.22 64.4–83.1	72.3 ±4.64 63.1–84.8	74.7 ±3.54 63.7–85.3	75.3 ±3.25 65.5–81.5
Haryanavi ♂ n=95–96, ♀ n=52	183.5 ±6.26 161–198	176.6 ±7.80 158–194	129.2 ±5.20 118–142	124.7 ±5.80 113–139	132.4 ±5.53 119–149	129.9 ±4.77 118–141	70.5 ±3.79 62.5–82.0	70.7 ±3.67 63.2–84.0	72.2 ±3.50 65.7–83.2	73.6 ±3.40 67.4–82.3
Hindi ♂ n=173–182, ♀ n=115–121	177.2 ±6.66 159–191	170.4 ±6.83 152–186	125.8 ±5.88 106–144	121.4 ±6.41 106–142	130.3 ±5.18 116–145	126.6 ±5.86 110–148	71.1 ±3.98 61.7–83.0	71.4 ±4.08 62.9–82.6	73.6 ±3.19 64.7–82.4	74.4 ±3.56 66.9–83.1
Urdu ♂ n=14, ♀ n=5–6	176.1 ±5.41 170–190	171.8 ±8.47 159–182	127.1 ±4.18 119–135	128.0 ±9.67 119–144	134.8 ±4.95 126–140	129.2 ±7.16 122–140	72.2 ±3.09 67.6–78.9	74.6 ±6.35 68.8–82.8	76.6 ±3.18 72.4–81.8	73.4 ±3.24 69.7–77.6

Konkani	178.6	163.8	128.9	122.3	135.3	129.0	72.2	75.1	75.8	81.1
♂ n=16–	±7.64	±12.97	±5.49	±5.85	±6.72	±2.00	±3.46	±8.32	±2.22	±5.24
20, ♀	165–	146–	120–	115–	126–	127–	65.4–	67.6–	71.2–	77.1–
n=3–4	190	176	140	127	149	131	78.2	87.0	79.8	87.0
Telugu	177.8	170.5	128.2	123.3	134.2	127.3	72.2	72.4	75.5	74.8
♂ n=74–	±6.85	±7.78	±7.21	±6.54	±6.02	±5.65	±4.59	±3.97	±3.47	±3.47
79, ♀	153–	153–	106–	105–	123–	119–	63.6–	66.3–	67.4–	67.4–
n=54–55	193	184	149	138	148	145	83.2	83.0	84.4	84.3
Kannada	173.0	166.2	125.8	121.8	132.3	128.0	72.9	73.4	76.6	77.1
♂ n=149,	±8.09	±6.06	±6.54	±6.67	±5.21	±5.05	±5.06	±4.58	±3.76	±3.40
♀ n=61–	158–	150–	109–	109–	117–	119–	61.2–	64.7–	66.8–	71.4–
62	192	183	147	139	147	142	86.5	84.1	88.0	86.6
Tulu	178.5	166.1	129.9	121.6	134.3	125.8	73.0	73.3	75.4	75.8
♂ n=31, ♀	±9.39	±5.86	±5.08	±7.24	±5.41	±5.56	±4.49	±4.19	±4.58	±3.22
n=19	159–	156–	118–	110–	122–	114–	64.6–	66.7–	66.8–	71.2–
	193	174	140	135	145	137	84.3	82.5	85.8	83.6
Tamil	178.0	170.5	127.3	121.7	133.8	129.3	71.7	71.5	75.3	76.0
♂ n=111–	±8.62	±7.15	±5.47	±6.26	±5.49	±5.41	±4.92	±4.31	±3.80	±3.41
116, ♀	154–	152–	114–	107–	119–	115–	62.2–	62.5–	65.1–	69.1–
n=65–71	196	187	142	138	150	142	83.8	85.4	85.1	85.4
Malayalam	177.2	171.1	126.2	123.6	134.6	126.3	71.3	72.4	76.9	73.5
♂ n=11–	±6.99	±8.83	±4.11	±6.96	±7.06	±4.35	±3.72	±5.32	±3.53	±2.49
13, ♀	166–	159–	119–	114–	119–	119–	64.7–	63.3–	68.0–	69.4–
n=10–12	191	183	132	141	144	132	78.9	81.5	80.3	77.2

Table S2. Indians' additional overall and parietal measurements of the cranial vault – means, standard deviations and ranges

Series	♂ NOL (mm)	♀ NOL (mm)	♂ AUB (mm)	♀ AUB (mm)	♂ PAC (mm)	♀ PAC (mm)	♂ PAS (mm)	♀ PAS (mm)	♂ PAF (mm)	♀ PAF (mm)
Punjabi	173.4	168.3	115.0	110.8	107.7	107.4	23.1	23.8	55.5	53.8
♂ n=100–119, ♀ n=59–71	±7.98	±7.05	±5.61	±5.24	±7.21	±7.19	±3.20	±3.46	±5.73	±6.08
	149–194	147–184	101–136	89–120	87–130	84–128	16–32	14–36	40–69	44–79
Haryanavi	180.3	173.8	118.7	115.8	110.7	109.1	23.2	22.6	56.2	55.9
♂ n=96, ♀ n=51–52	±6.59	±7.79	±4.57	±4.01	±7.17	±5.42	±2.66	±2.26	±4.87	±4.52
	154–196	154–191	100–130	105–126	90–139	92–125	17–29	18–29	43–67	44–65
Hindi	174.1	167.7	115.6	109.8	109.6	106.2	23.6	22.5	55.5	53.0
♂ n=173–182, ♀ n=115–121	±6.58	±6.70	±4.95	±5.97	±6.67	±6.99	±3.05	±3.41	±5.58	±4.85
	158–190	149–183	101–127	92–123	81–129	83–119	15–31	14–30	40–73	40–65
Urdu	174.0	168.2	116.1	110.5	111.2	108.5	26.1	23.3	58.2	55.8
♂ n=14, ♀ n=6	±5.60	±7.66	±3.03	±5.74	±5.80	±6.19	±3.25	±2.25	±4.81	±7.28
	167–188	156–179	111–120	103–118	102–121	100–119	20–32	21–26	50–68	46–65
Konkani	175.8	163.0	116.6	110.8	109.6	102.0	24.6	22.0	55.9	44.7
♂ n=16–	±7.68	±7.66	±4.81	±4.65	±5.97	±19.00	±3.71	±4.00	±7.48	±9.81

20, ♀ n=3–4	161–188	144–174	107–127	105–115	102–121	81–118	20–33	18–26	39–68	39–56
Telugu ♂ n=72–79, ♀ n=54–55	174.4 ±6.57 152–190	167.7 ±7.66 152–182	116.9 ±5.71 96–129	110.5 ±6.22 93–125	111.1 ±6.49 94–126	106.0 ±6.02 92–117	24.6 ±2.93 18–33	23.0 ±3.57 11–31	57.8 ±4.90 42–67	55.5 ±5.66 39–69
Kannada ♂ n=149, ♀ n=61–62	170.1 ±7.76 156–190	164.0 ±6.05 149–182	114.9 ±5.29 103–129	110.2 ±4.64 95–119	108.6 ±7.41 86–125	105.0 ±7.00 87–122	24.3 ±3.24 15–33	23.6 ±3.70 12–33	53.5 ±6.46 33–70	52.3 ±5.44 38–65
Tulu ♂ n=31, ♀ n=19	176.0 ±8.97 158–190	163.9 ±6.55 154–173	117.8 ±4.88 106–127	108.6 ±5.93 99–121	110.6 ±7.86 96–124	104.1 ±4.42 92–112	25.2 ±3.78 16–31	23.2 ±2.80 18–28	57.1 ±4.94 42–65	54.6 ±5.55 45–62
Tamil ♂ n=112–116, ♀ n=66–71	174.7 ±8.28 153–193	167.6 ±7.07 151–183	116.3 ±4.29 108–132	110.3 ±5.71 99–128	110.9 ±7.08 95–125	107.6 ±7.50 87–125	24.1 ±3.03 17–31	23.5 ±3.82 15–34	53.3 ±5.95 32–67	51.7 ±5.58 40–65
Malayalam ♂ n=11–13, ♀ n=10–12	174.0 ±6.36 165–183	169.0 ±8.59 156–179	115.9 ±4.29 109–121	111.3 ±4.25 106–119	107.8 ±6.13 98–118	108.0 ±7.85 94–117	22.5 ±2.73 18–27	23.4 ±3.53 17–28	53.9 ±4.01 50–62	55.5 ±4.43 45–60

Table S3. Indians' measurements of the frontal vault – means, standard deviations and ranges

Series	♂ STB (mm)	♀ STB (mm)	♂ XFB (mm)	♀ XFB (mm)	♂ FRC (mm)	♀ FRC (mm)	♂ FRS (mm)	♀ FRS (mm)	♂ FRF (mm)	♀ FRF (mm)
Punjabi ♂ n=100–115, ♀ n=59–69	108.6	108.5	112.5	110.7	108.8	106.8	26.3	27.2	50.9	49.5
	±6.53	±5.61	±6.11	±5.74	±5.85	±5.50	±2.71	±3.11	±6.21	±6.38
	93–127	93–122	97–127	97–126	93–123	94–122	20–34	20–36	36–70	36–67
Haryanavi ♂ n=95–96, ♀ n=51–52	111.1	109.3	114.1	110.8	111.4	108.1	26.5	26.9	53.5	51.4
	±4.85	±4.97	±4.44	±4.24	±4.18	±5.13	±2.70	±3.19	±4.38	±4.62
	95–124	99–119	101–126	101–121	100–124	93–125	19–34	20–41	44–68	38–60
Hindi ♂ n=173–182, ♀ n=115–121	109.0	107.2	112.3	109.8	108.9	105.7	26.1	27.3	51.3	48.1
	±5.92	±5.89	±5.52	±5.76	±4.72	±4.90	±2.82	±2.90	±5.00	±4.85
	89–127	91–124	97–135	94–126	96–120	92–118	18–36	20–37	37–63	35–60
Urdu ♂ n=14, ♀ n=6	114.4	110.3	115.9	112.0	112.4	107.8	26.8	26.7	55.3	55.5
	±6.39	±5.19	±7.15	±5.45	±5.33	±5.85	±2.67	±3.20	±3.93	±8.98
	105–128	106–118	106–130	107–119	101–119	100–114	23–31	23–31	49–62	47–72
Konkani ♂ n=16–20, ♀ n=3–4	112.7	110.5	116.1	109.0	114.3	100.7	27.4	24.3	55.2	46.7
	±5.99	±5.19	±5.39	±5.45	±6.74	±3.21	±3.50	±2.52	±6.56	±6.66
	101–124	109–114	101–126	100–117	105–126	97–103	20–34	22–27	44–67	39–51

Telugu ♂ n=72–79, ♀ n=55	112.3 ±6.05 99–134	107.7 ±5.19 92–118	115.2 ±7.18 100–141	110.1 ±5.45 96–122	111.3 ±5.12 100–124	105.9 ±6.13 94–124	27.2 ±2.97 21–35	27.3 ±3.14 21–34	55.0 ±4.69 45–68	50.3 ±5.23 41–66
Kannada ♂ n=148–149, ♀ n=61–62	111.0 ±6.54 95–132	107.4 ±5.88 94–123	115.5 ±6.35 101–134	111.9 ±5.57 101–126	109.3 ±5.32 94–130	106.1 ±5.61 94–121	26.2 ±3.22 16–37	26.7 ±2.67 22–35	51.5 ±5.56 39–65	49.2 ±6.07 37–64
Tulu ♂ n=31, ♀ n=19	115.5 ±5.75 104–128	107.9 ±6.00 99–120	119.1 ±5.75 109–134	110.2 ±7.17 99–125	112.6 ±4.84 103–121	106.5 ±5.19 98–121	27.2 ±3.32 21–34	28.3 ±2.45 24–32	54.8 ±4.37 48–65	50.6 ±5.37 42–61
Tamil ♂ n=112–115, ♀ n=66–71	112.0 ±5.70 99–126	108.9 ±5.44 99–123	115.5 ±5.58 102–132	111.9 ±5.71 103–128	111.2 ±5.01 102–126	107.4 ±5.24 95–125	27.1 ±3.03 20–34	27.7 ±2.95 19–35	51.1 ±4.47 40–64	47.9 ±5.85 39–70
Malayalam ♂ n=11–13, ♀ n=10–12	111.5 ±4.07 102–118	109.3 ±5.69 101–117	114.0 ±2.68 109–118	111.4 ±5.81 104–123	111.3 ±4.27 103–117	105.7 ±5.60 96–112	26.6 ±1.43 25–30	26.3 ±2.16 23–29	53.3 ±2.33 50–56	48.4 ±4.74 42–55

Table S4. Indians’ cranial measurements involving the occipital bone – means, standard deviations and ranges

Series	♂ ASB (mm)	♀ ASB (mm)	♂ OCC (mm)	♀ OCC (mm)	♂ OCS (mm)	♀ OCS (mm)	♂ OCF (mm)	♀ OCF (mm)	♂ FOL (mm)	♀ FOL (mm)
Punjabi ♂ n=106–119, ♀ n=63–71	102.5<br>±5.52<br>91–114	99.8<br>±5.72<br>86–115	93.4<br>±6.37<br>78–112	91.4<br>±6.26<br>78–109	26.6<br>±4.01<br>18–36	25.1<br>±4.12<br>17–38	52.1<br>±8.40<br>37–70	45.1<br>±6.42<br>30–63	34.7<br>±2.84<br>27–44	33.9<br>±2.28<br>29–39
Haryanavi ♂ n=94–96, ♀ n=51–52	106.5<br>±6.25<br>91–119	102.4<br>±4.99<br>91–113	94.6<br>±6.01<br>81–111	91.8<br>±5.65<br>79–110	28.7<br>±3.97<br>20–41	26.8<br>±3.47<br>18–36	50.9<br>±7.75<br>37–76	46.7<br>±7.88<br>31–67	35.7<br>±2.72<br>28–42	34.7<br>±2.77<br>29–40
Hindi ♂ n=175–182, ♀ n= 117–121	102.2<br>±4.89<br>90–114	99.2<br>±4.86<br>88–111	91.9<br>±5.23<br>78–109	90.1<br>±6.51<br>77–116	27.1<br>±3.76<br>19–37	26.3<br>±3.97<br>19–37	48.1<br>±6.99<br>31–70	44.8<br>±7.08<br>28–65	34.7<br>±2.60<br>27–42	34.5<br>±2.89<br>27–41
Urdu ♂ n=14, ♀ n=5–6	103.5<br>±5.46<br>97–114	103.7<br>±4.08<br>100–111	93.1<br>±4.63<br>83–100	91.6<br>±7.16<br>82–100	25.6<br>±2.95<br>20–30	23.6<br>±5.86<br>17–33	50.6<br>±6.86<br>38–60	44.6<br>±12.0<br>31–58	33.5<br>±3.37<br>25–39	33.5<br>±3.01<br>31–38
Konkani ♂ n=16–20, ♀ n=3–4	103.2<br>±6.43<br>94–119	104.7<br>±7.51<br>97–112	94.8<br>±7.63<br>82–109	91.8<br>±7.18<br>83–99	25.6<br>±4.26<br>17–34	22.3<br>±0.50<br>17–27	53.3<br>±9.48<br>40–71	44.0<br>±8.49<br>33–56	34.5<br>±2.61<br>29–40	34.6<br>±2.88<br>28–37

Telugu<br>♂ n=77–79, ♀ n=53–55	103.2<br>±5.32<br>92–119	99.1<br>±4.61<br>87–112	93.8<br>±5.89<br>79–110	91.7<br>±6.61<br>80–110	26.7<br>±4.39<br>18–35	26.2<br>±4.80<br>18–39	50.4<br>±8.66<br>36–72	46.5<br>±6.52<br>31–62	34.5<br>±2.23<br>30–40	33.5<br>±3.01<br>22–41
Kannada<br>♂ n=149, ♀ n=61–62	101.2<br>±5.07<br>90–121	98.4<br>±4.25<br>89–110	91.5<br>±5.59<br>76–110	89.0<br>±6.18<br>72–103	24.8<br>±3.85<br>16–38	23.5<br>±3.34<br>15–32	46.4<br>±7.58<br>29–69	43.5<br>±5.69<br>31–54	34.5<br>±3.02<br>24–45	33.3<br>±2.47<br>28–40
Tulu<br>♂ n=31, ♀ n=19	104.0<br>±5.49<br>93–113	97.5<br>±6.01<br>85–107	96.8<br>±7.26<br>86–111	88.5<br>±6.10<br>80–104	25.5<br>±5.32<br>18–41	22.9<br>±3.54<br>17–32	53.3<br>±8.84<br>38–78	40.9<br>±6.24<br>32–57	34.5<br>±3.53<br>28–45	32.4<br>±2.81<br>26–38
Tamil<br>♂ n=111–116, ♀ n=68–71	104.1<br>±4.88<br>94–120	99.3<br>±5.29<br>86–113	93.5<br>±5.03<br>84–112	91.5<br>±5.85<br>81–106	26.9<br>±3.50<br>19–34	24.7<br>±3.96<br>17–36	48.9<br>±7.53<br>33–66	43.2<br>±5.89<br>30–58	35.0<br>±2.79<br>29–42	34.1<br>±2.43<br>29–41
Malayalam<br>♂ n=11–13, ♀ n=10–12	103.6<br>±2.75<br>98–107	101.8<br>±6.26<br>95–119	95.1<br>±8.51<br>80–110	89.8<br>±6.91<br>76–101	25.0<br>±4.97<br>18–36	24.7<br>±3.95<br>20–33	49.6<br>±7.75<br>32–60	43.6<br>±8.09<br>34–60	34.8<br>±2.49<br>32–39	33.8<br>±1.19<br>32–36

Table S5. Indians' mastoid process measurements and cranial chord indices – means, standard deviations and ranges

Series	♂<br>MDH<br>(mm)	♀<br>MDH<br>(mm)	♂<br>MDB<br>(mm)	♀<br>MDB<br>(mm)	♂<br>FRC:<br>FRS	♀<br>FRC:<br>FRS	♂<br>PAC:<br>PAS	♀<br>PAC:<br>PAS	♂<br>OCC:<br>OCS	♀<br>OCC:<br>OCS
Punjabi<br>♂ n=100–119, ♀ n=63–71	27.5<br>±3.99<br>18–40	24.1<br>±4.61<br>11–39	10.3<br>±2.38<br>4–16	9.0<br>±2.48<br>3–18	24.1<br>±2.11<br>19.4–30.5	25.4<br>±2.51<br>18.9–31.9	21.4<br>±2.38<br>15.6–27.4	22.1<br>±2.69<br>16.7–34.0	28.4<br>±3.43<br>20.9–35.4	27.5<br>±3.83<br>18.5–36.4
Haryanavi<br>♂ n=96, ♀ n=51–52	29.6<br>±3.82<br>21–39	27.4<br>±3.81<br>20–36	11.4<br>±2.20<br>7–17	10.0<br>±1.97<br>6–14	23.8<br>±2.12<br>18.3–29.6	24.9<br>±3.00<br>18.6–39.0	21.0<br>±2.07<br>16.8–27.9	20.8<br>±1.93<br>17.1–27.5	30.3<br>±3.66<br>18.2–37.2	29.2<br>±3.71<br>20.2–37.1
Hindi<br>♂ n=170–182, ♀ n= 115–119	27.6<br>±3.39<br>18–36	24.3<br>±3.99<br>12–34	11.2<br>±2.39<br>5–20	9.0<br>±2.39<br>4–15	24.0<br>±2.28<br>17.1–33.6	25.8<br>±2.29<br>20.2–34.3	21.5<br>±2.34<br>13.6–28.0	21.1<br>±2.45<br>15.4–27.4	29.4<br>±3.65<br>19.6–37.6	29.2<br>±3.71<br>21.2–40.2
Urdu<br>♂ n=13–14, ♀ n=5–6	24.9<br>±3.15<br>19–30	21.2<br>±1.73<br>16–26	11.4<br>±2.18<br>8–15	10.7<br>±5.17<br>6–20	23.8<br>±1.86<br>20.5–26.7	24.7<br>±2.15<br>22.4–27.2	23.3<br>±2.37<br>18.5–27.8	21.5<br>±1.43<br>19.8–24.1	27.5<br>±2.80<br>21.9–31.0	25.6<br>±4.69<br>20.7–33.0
Konkani<br>♂ n=16–20, ♀ n=3–4	25.6<br>±3.38<br>20–32	22.3<br>±2.99<br>19–26	12.3<br>±2.83<br>8–18	7.5<br>±1.73<br>5–9	23.9<br>±2.27<br>19.0–27.9	24.1<br>±1.84<br>22.7–26.2	22.4<br>±2.53<br>18.0–28.4	21.7<br>±2.86<br>18.6–24.3	26.9<br>±3.83<br>20.7–35.8	24.4<br>±2.12<br>22.2–26.5

Telugu ♂ n=72–78, ♀ n=55	25.2 ±4.05 15–33	21.0 ±4.26 12–31	11.5 ±2.42 7–18	8.5 ±2.62 3–14	24.4 ±2.21 20.2–32.1	25.8 ±2.22 21.6–31.2	22.1 ±2.17 16.8–27.7	21.6 ±2.79 11.8–28.3	28.4 ±3.98 18.8–36.7	28.5 ±3.91 20.2–37.4
Kannada ♂ n=149, ♀ n=61–62	28.1 ±3.97 18–36	24.9 ±4.12 14–34	11.0 ±2.44 5–19	9.1 ±2.55 3–17	24.0 ±2.48 14.0–32.4	25.1 ±2.04 19.1–28.9	22.4 ±2.72 15.2–34.9	22.4 ±2.74 12.4–29.8	27.1 ±3.74 18.4–37.6	26.4 ±3.20 19.7–36.3
Tulu ♂ n=31, ♀ n=19	24.3 ±3.20 17–32	20.1 ±3.42 15–25	10.9 ±2.52 6–17	8.0 ±2.26 4–13	24.1 ±2.38 19.1–29.3	26.6 ±2.01 23.1–30.6	22.7 ±2.47 16.5–26.7	22.3 ±2.57 17.3–26.9	26.2 ±4.28 20.0–37.3	25.8 ±3.16 20.0–30.9
Tamil ♂ n=111–116, ♀ n=66–71	25.0 ±3.22 18–34	21.2 ±3.78 14–31	11.2 ±2.38 7–20	9.6 ±2.66 4–17	24.3 ±2.26 18.8–31.7	25.7 ±2.25 19.0–31.5	21.8 ±2.44 15.5–29.0	21.7 ±2.51 17.0–27.2	28.8 ±3.25 22.3–36.7	26.9 ±3.56 20.2–34.6
Malayalam ♂ n=11–13, ♀ n=10–12	24.6 ±3.04 18–30	22.0 ±3.10 18–28	12.0 ±2.74 8–17	10.9 ±2.02 8–15	24.0 ±1.22 22.3–25.6	24.9 ±1.63 23.5–28.4	20.9 ±1.88 17.3–23.6	21.6 ±2.18 17.5–25.5	26.2 ±3.74 21.1–32.7	27.5 ±3.79 22.8–35.1

Table S6. Indians' measurements and indices showing vault-face relationships – means, standard deviations and ranges

Series	♂ BNL (mm)	♀ BNL (mm)	♂ BPL (mm)	♀ BPL (mm)	♂ BNL: BPL	♀ BNL: BPL	♂ XCB: ZYB	♀ XCB: ZYB	♂ ZYB: ASB	♀ ZYB: ASB
Punjabi	99.4	94.9	94.3	90.0	95.0	94.7	96.8	95.3	83.5	84.9
♂ n=115–	±4.56	±5.05	±5.04	±6.18	±4.08	±4.76	±4.68	±5.18	±4.13	±5.02
119, ♀	86–	83–	82–	75–	86.0–	85.9–	84.4–	82.4–	71.0–	72.0–
n=67–70	113	106	106	107	106.6	109.1	109.2	108.8	92.9	98.1
Haryanavi	101.7	99.0	95.2	93.6	93.8	94.6	98.5	98.3	83.8	83.8
♂ n=93–	±5.51	±4.92	±5.02	±5.16	±5.13	±4.56	±4.17	±4.03	±5.46	±3.96
96, ♀	91–	86–	84–	81–	77.7–	85.4–	87.4–	89.8–	70.8–	74.2–
n=51–52	118	115	116	105	114.9	102.1	107.5	110.3	98.2	93.3
Hindi	98.7	93.8	94.5	90.1	95.8	96.1	98.8	96.2	82.4	85.0
♂ n=180–	±4.01	±5.20	±5.01	±5.52	±4.66	±5.33	±4.18	±4.53	±4.25	±5.11
182, ♀ n=	86–	80–	80–	74–	82.5–	86.0–	86.2–	83.3–	69.5–	74.0–
115–121	108	105	108	107	112.0	113.3	113.2	104.4	91.4	104.1
Urdu	101.1	98.6	93.9	91.6	93.0	93.1	98.8	93.3	82.5	88.1
♂ n=14, ♀	±6.00	±9.34	±5.38	±7.64	±5.33	±5.99	±4.56	±5.77	±4.25	±5.22
n=5–6	82–	87–	85–	84–	85.3–	86.4–	90.2–	86.3–	75.4–	82.7–
	107	110	103	103	103.7	98.1	104.0	100.8	90.8	94.3
Konkani	101.9	96.5	96.0	89.8	94.3	93.0	98.0	96.0	82.3	88.9
♂ n=19–	±5.61	±8.35	±6.16	±8.06	±4.28	±2.74	±3.42	±4.76	±4.97	±8.13
20, ♀	90–	92–	86–	83–	86.0–	90.2–	94.0–	90.6–	75.6–	84.0–
n=3–4	114	109	106	101	101.9	96.8	105.0	99.1	93.3	98.2

Telugu	99.5	93.5	94.9	88.8	95.5	95.0	98.0	95.5	82.3	85.0
♂ n=78–	±5.06	±5.60	±4.67	±5.44	±4.64	±5.48	±4.35	±5.18	±4.34	±5.35
79, ♀	83–	79–	83–	78–	86.0–	79.8–	84.3–	83.1–	73.3–	73.7–
n=53–55	111	104	107	102	108.1	107.0	107.8	105.4	99.2	98.0
Kannada	98.3	94.3	92.2	88.9	93.9	94.5	97.6	95.6	82.6	84.7
♂ n=146–	±4.84	±4.13	±5.27	±4.43	±4.58	±5.02	±4.26	±4.17	±4.74	±3.90
149, ♀	84–	85–	82–	77–	82.8–	78.3–	83.7–	82.7–	72.0–	74.4–
n=60–62	115	107	105	101	108.3	105.2	109.9	105.5	96.3	96.3
Tulu	100.9	93.5	94.2	88.4	93.4	94.5	96.7	94.2	83.0	85.5
♂ n=30–	±5.28	±5.27	±5.31	±6.15	±4.89	±5.77	±4.89	±4.83	±5.17	±7.20
31, ♀	87–	84–	85–	79–	86.1–	84.0–	84.1–	82.0–	70.9–	69.0–
n=18–19	110	106	109	100	104.8	106.6	106.5	100.8	92.4	97.1
Tamil	99.6	93.9	94.5	88.7	95.0	94.6	98.4	96.3	83.1	85.1
♂ n=111–	±4.97	±5.19	±5.49	±5.42	±4.76	±4.98	±4.32	±5.11	±4.18	±5.29
116, ♀	83–	79–	81–	74–	81.8–	85.3–	87.4–	86.3–	72.9–	95.4–
n=69–71	113	105	106	101	111.9	106.3	107.3	106.7	95.2	101.0
Malayalam	98.8	95.3	96.6	92.3	97.8	97.1	99.3	95.9	82.7	86.1
♂ n=11–	±5.37	±3.72	±5.50	±3.20	±4.68	±4.80	±4.20	±4.85	±4.89	±5.59
13, ♀	88–	89–	85–	87–	89.3–	88.8–	93.6–	85.8–	73.7–	77.9–
n=12	107	100	106	97	106.6	105.6	108.0	103.4	89.7	98.3

Table S7. Indians' main facial measurements and indices – means, standard deviations and ranges

Series	♂ ZYB (mm)	♀ ZYB (mm)	♂ JUB (mm)	♀ JUB (mm)	♂ NPH (mm)	♀ NPH (mm)	♂ ZYB: NPH	♀ ZYB: NPH	♂ ZYB: ZMB	♀ ZYB: ZMB
Punjabi ♂ n=116–119, ♀ n=65–70	123.2<br>±6.06<br>108–135	117.8<br>±5.56<br>97–130	109.6<br>±5.17<br>96–124	104.4<br>±5.73<br>79–118	63.6<br>±5.19<br>49–76	59.2<br>±5.35<br>46–69	51.6<br>±3.69<br>40.5–59.7	50.5<br>±3.92<br>37.1–57.1	75.0<br>±3.15<br>65.6–84.1	75.4<br>±4.06<br>62.1–87.0
Haryanavi ♂ n=95–96, ♀ n=51–52	127.2<br>±4.55<br>114–137	122.4<br>±4.11<br>114–129	110.9<br>±4.22<br>102–120	108.0<br>±5.83<br>92–124	64.7<br>±4.84<br>53–77	62.5<br>±5.80<br>49–74	50.9<br>±4.02<br>42.7–61.9	51.1<br>±4.74<br>38.6–60.8	72.3<br>±3.85<br>63.0–82.0	73.8<br>±4.60<br>63.5–81.8
Hindi ♂ n=179–181, ♀ n= 115–119	124.1<br>±4.95<br>109–134	117.1<br>±7.11<br>95–130	109.6<br>±4.52<br>99–124	103.7<br>±6.25<br>83–124	64.0<br>±4.57<br>52–78	60.7<br>±5.40<br>49–75	51.5<br>±3.56<br>40.3–64.5	51.9<br>±4.10<br>42.7–62.8	74.5<br>±3.50<br>65.1–85.5	76.2<br>±3.52<br>67.3–85.0
Urdu ♂ n=14, ♀ n=5–6	125.4<br>±4.57<br>119–134	116.4<br>±8.79<br>106–127	112.6<br>±5.08<br>106–124	106.0<br>±7.67<br>96–115	64.8<br>±3.96<br>56–70	58.0<br>±3.90<br>53–63	51.7<br>±2.75<br>46.7–58.0	49.1<br>±2.89<br>45.2–53.1	75.4<br>±4.15<br>68.0–82.6	76.9<br>±2.08<br>74.0–79.6
Konkani ♂ n=18–20, ♀ n=3–4	125.9<br>±5.38<br>116–138	118.0<br>±6.08<br>114–125	111.1<br>±4.41<br>103–119	103.0<br>±5.72<br>96–110	63.5<br>±4.51<br>55–74	63.3<br>±7.14<br>55–72	50.6<br>±3.63<br>44.6–58.6	53.0<br>±4.91<br>47.8–57.6	75.0<br>±3.56<br>63.6–79.5	74.3<br>±1.98<br>74.6–78.4

Telugu	125.5	117.8	111.1	104.6	63.2	58.4	50.4	49.5	74.6	74.3
♂ n=78–	±5.35	±7.61	±4.81	±6.67	±4.67	±5.35	±3.84	±3.35	±3.38	±3.54
79, ♀	110–	96–	101–	86–	51–	46–	40.6–	43.1–	68.7–	66.9–
n=53–55	138	133	124	126	72	70	62.7	55.7	82.3	82.6
Kannada	122.6	116.2	108.8	104.0	62.9	59.5	51.3	51.2	75.2	75.8
♂ n=148–	±5.51	±4.16	±4.47	±3.28	±4.76	±4.90	±3.97	±3.89	±3.47	±2.82
149, ♀	108–	107–	95–	95–	52–	47–	43.6–	42.3–	65.1–	68.0–
n=61–62	136	126	119	111	74	72	64.8	58.5	88.0	81.2
Tulu	125.6	114.8	111.6	102.8	61.7	54.6	49.3	47.6	74.3	74.8
♂ n=31, ♀	±7.14	±7.97	±4.64	±5.87	±4.32	±5.51	±4.17	±3.11	±4.81	±3.80
n=18–19	106–	101–	101–	91–	50–	43–	40.0–	41.3–	62.7–	67.6–
	135	127	120	113	69	63	56.4	52.5	90.6	84.4
Tamil	125.2	117.2	109.8	104.7	63.4	58.2	50.7	49.9	74.3	75.7
♂ n=102–	±4.52	±6.81	±4.17	±6.56	±4.21	±4.83	±3.65	±3.11	±3.10	±3.13
114, ♀	115–	101–	101–	87–	54–	48–	40.5–	43.4–	67.5–	66.7–
n=64–71	139	136	122	120	72	68	60.2	56.8	83.1	83.8
Malayalam	125.5	118.3	111.0	105.2	61.6	57.9	49.2	49.0	73.3	75.2
♂ n=11–	±5.37	±4.60	±4.24	±2.49	±2.90	±3.96	±2.46	±2.97	±2.47	±2.80
13, ♀	117–	113–	107–	101–	55–	49–	45.1–	43.4–	66.9–	67.8–
n=10–12	136	127	123	108	65	65	53.3	53.8	75.4	78.0

Table S8. Indians’ frontal-facial measurements and index of upper facial projection – means, standard deviations and ranges

Series	♂ FMB (mm)	♀ FMB (mm)	♂ NAS (mm)	♀ NAS (mm)	♂ FMB: NAS	♀ FMB: NAS	♂ GLS (mm)	♀ GLS (mm)	♂ SOS (mm)	♀ SOS (mm)
Punjabi<br>♂ n=119, ♀ n=71	94.9<br>±4.52<br>86–108	91.2<br>±4.23<br>74–102	19.4<br>+2.87<br>12–29	18.4<br>+2.78<br>9–24	20.4<br>±2.95<br>12.9–31.5	20.2<br>±3.01<br>9.4–26.1	3.3<br>±1.20<br>1–6	2.4<br>±1.10<br>1–5	5.9<br>±2.20<br>2–11	4.1<br>±1.82<br>2–10
Haryanavi<br>♂ n=95–96, ♀ n=52	95.2<br>±3.83<br>86–106	92.5<br>±3.68<br>85–105	20.2<br>+2.67<br>14–28	19.8<br>+2.08<br>15–23	21.2<br>±2.58<br>14.9–30.4	21.3<br>±2.05<br>17.0–25.9	3.4<br>±1.24<br>1–6	2.2<br>±1.12<br>1–5	3.9<br>±1.47<br>1–10	3.4<br>±1.69<br>1–8
Hindi<br>♂ n=180–182, ♀ n= 119–120	94.0<br>±3.83<br>84–110	89.7<br>±4.95<br>76–102	19.4<br>+2.74<br>13–29	18.3<br>+2.57<br>12–25	20.6<br>±2.78<br>14.3–30.9	20.4<br>±2.69<br>13.6–26.3	3.4<br>±1.30<br>1–9	2.1<br>±1.03<br>0–7	5.0<br>±2.02<br>1–10	3.3<br>±1.80<br>0–11
Urdu<br>♂ n=14, ♀ n=6	96.2<br>±4.21<br>89–103	91.5<br>±6.22<br>84–101	20.8<br>+2.04<br>17–23	18.7<br>+2.25<br>17–23	21.6<br>±2.29<br>18.0–25.8	20.4<br>±1.46<br>18.9–22.8	3.2<br>±1.88<br>1–5	1.8<br>±0.98<br>1–3	3.6<br>±0.84<br>3–6	2.2<br>±0.75<br>1–3
Konkani<br>♂ n=20, ♀ n=4	95.8<br>±3.27<br>91–101	94.3<br>±4.86<br>88–99	20.3<br>+2.02<br>15–23	19.5<br>+3.32<br>15–22	21.2<br>±2.21<br>16.3–24.2	20.8<br>±3.84<br>15.2–23.7	3.0<br>±0.83<br>1–4	1.8<br>±1.75<br>0–4	2.6<br>±0.69<br>1–4	2.3<br>±2.25<br>1–4

Telugu<br>♂ n=76–79,<br>♀ n=53–55	95.2<br>±3.98<br>86–<br>103	90.5<br>±4.99<br>78–<br>100	19.4<br>+2.95<br>11–26	18.5<br>+2.95<br>11–25	20.3<br>±2.78<br>12.4–<br>25.2	20.5<br>±2.85<br>11.3–<br>25.8	3.3<br>±1.22<br>1–8	1.7<br>±1.00<br>0–5	3.5<br>±1.01<br>2–8	2.2<br>±0.94<br>1–4
Kannada<br>♂ n=149, ♀<br>n=61–62	95.0<br>±4.11<br>83–<br>111	91.3<br>±3.39<br>82–97	18.6<br>+2.86<br>8–26	18.6<br>+2.36<br>13–25	19.6<br>±2.74<br>9.2–<br>26.5	20.4<br>±2.32<br>14.9–<br>26.7	2.5<br>±1.27<br>0–8	1.2<br>±0.70<br>0–3	2.1<br>±0.99<br>0–5	1.4<br>±0.66<br>0–3
Tulu<br>♂ n=31, ♀<br>n=19	97.0<br>±3.83<br>89–<br>104	89.5<br>±5.06<br>81–99	20.2<br>+2.78<br>15–25	18.9<br>+2.30<br>13–22	20.8<br>±2.57<br>15.2–<br>26.3	21.2<br>±2.42<br>14.8–<br>24.4	2.7<br>±1.10<br>0–5	1.5<br>±0.70<br>1–3	2.9<br>±0.85<br>1–5	1.7<br>±0.89<br>0–3
Tamil<br>♂ n=116, ♀<br>n=71	95.1<br>±3.89<br>86–<br>106	90.6<br>±4.51<br>78–<br>100	19.6<br>+2.44<br>14–26	18.2<br>+2.43<br>13–27	20.6<br>±2.30<br>15.4–<br>27.1	20.1<br>±2.73<br>13.8–<br>31.0	3.1<br>±1.02<br>1–6	1.7<br>±0.95<br>0–4	2.7<br>±1.42<br>1–12	2.2<br>±1.42<br>0–10
Malayalam<br>♂ n=13, ♀<br>n=12	95.6<br>±2.02<br>92–99	92.7<br>±1.97<br>89–96	18.5<br>+2.44<br>13–22	18.1<br>+1.98<br>15–22	19.3<br>±2.53<br>13.1–<br>23.2	19.5<br>±2.07<br>16.3–<br>23.4	3.1<br>±0.95<br>1–4	2.0<br>±1.28<br>0–4	2.8<br>±0.7<br>3<br>2–4	1.7<br>±1.1<br>5<br>0–3

Table S9. Indians' mid-facial breadths, orbital diameters and orbital index – means, standard deviations and ranges

Series	♂ EKB (mm)	♀ EKB (mm)	♂ ZMB (mm)	♀ ZMB (mm)	♂ OBH (mm)	♀ OBH (mm)	♂ OBB (mm)	♀ OBB (mm)	♂ OBB: OBH	♀ OBB: OBH
Punjabi ♂ n=119, ♀ n=70–71	94.7<br>±4.51<br>84–109	91.4<br>±3.88<br>74–100	92.6<br>±5.20<br>79–108	88.9<br>±5.50<br>77–104	33.1<br>±2.08<br>29–40	32.7<br>±2.12<br>28–37	39.2<br>±2.05<br>34–45	38.3<br>±2.05<br>31–43	84.6<br>±5.91<br>69.7–100.0	85.6<br>±6.19<br>71.4–100.0
Haryanavi ♂ n=96, ♀ n=52	94.4<br>±3.37<br>86–104	91.8<br>±3.74<br>83–103	91.9<br>±5.40<br>80–106	90.2<br>±5.19<br>75–99	33.4<br>±2.21<br>26–38	32.9<br>±2.63<br>25–39	39.4<br>±2.17<br>34–44	38.3<br>±2.17<br>32–44	84.8<br>±5.94<br>70.7–97.4	85.7<br>±7.07<br>64.1–103.1
Hindi ♂ n=180–182, ♀ n= 119–120	93.7<br>±3.37<br>85–104	89.8<br>±4.53<br>76–100	92.5<br>±4.58<br>79–104	88.4<br>±5.93<br>72–104	32.2<br>±1.90<br>27–37	31.8<br>±2.00<br>26–36	39.1<br>±1.69<br>35–44	37.5<br>±2.03<br>32–44	82.7<br>±4.75<br>69.8–95.6	84.7<br>±5.04<br>71.8–98.3
Urdu ♂ n=14, ♀ n=5–6	95.9<br>±4.38<br>88–105	91.5<br>±6.02<br>83–100	94.5<br>±4.47<br>85–101	89.2<br>±5.31<br>82–96	33.0<br>±1.80<br>30–36	33.2<br>±2.17<br>30–36	42.2<br>±2.08<br>38–46	38.8<br>±2.64<br>36–42	78.3<br>±4.93<br>71.4–85.4	84.3<br>±9.13<br>81.0–89.2
Konkani ♂ n=19–20, ♀ n=4	95.6<br>±3.74<br>90–104	94.5<br>±4.51<br>88–98	94.4<br>±5.13<br>82–103	90.5<br>±5.80<br>85–98	32.0<br>±2.94<br>27–39	33.5<br>±1.91<br>31–35	42.4<br>±1.90<br>39–45	40.5<br>±4.93<br>35–47	75.5<br>±5.73<br>67.5–92.9	83.4<br>±5.11<br>74.5–94.3

Telugu<br>♂ n=78–79, ♀ n=54–55	94.8<br>±3.69<br>87–104	90.9<br>±4.70<br>77–100	93.5<br>±5.15<br>83–108	87.4<br>±6.10<br>68–98	32.3<br>±1.70<br>28–36	32.0<br>±2.15<br>28–38	41.9<br>±2.31<br>37–47	40.3<br>±2.27<br>35–45	77.2<br>±5.60<br>66.7–91.9	79.6<br>±5.11<br>71.1–92.7
Kannada<br>♂ n=149, ♀ n=61–62	93.8<br>±3.75<br>81–103	90.1<br>±3.40<br>83–97	92.2<br>±4.79<br>80–105	88.1<br>±3.82<br>78–98	32.3<br>±2.06<br>28–38	32.3<br>±2.24<br>28–37	40.2<br>±2.78<br>33–46	38.8<br>±2.09<br>33–44	80.5<br>±5.60<br>69.0–94.4	83.4<br>±5.87<br>69.0–94.9
Tulu<br>♂ n=31, ♀ n=18–19	95.6<br>±4.92<br>82–104	90.1<br>±4.83<br>80–97	93.1<br>±5.23<br>84–107	85.8<br>±6.33<br>71–95	32.6<br>±2.20<br>29–38	31.7<br>±2.19<br>29–36	41.8<br>±1.76<br>38–44	39.4<br>±2.93<br>34–44	78.2<br>±5.63<br>70.5–92.3	80.6<br>±5.00<br>73.8–88.6
Tamil<br>♂ n=108–114, ♀ n=68–71	94.4<br>±3.63<br>86–105	90.4<br>±4.28<br>78–100	92.8<br>±5.26<br>80–105	88.4<br>±6.00<br>73–100	32.2<br>±1.84<br>27–37	31.9<br>±1.82<br>28–36	41.6<br>±2.06<br>37–46	39.8<br>±1.99<br>35–43	77.3<br>±4.83<br>68.2–92.3	80.1<br>±4.34<br>70.7–91.9
Malayalam<br>♂ n=11–12, ♀ n=11–12	94.7<br>±3.07<br>91–101	91.8<br>±1.99<br>88–95	91.8<br>±3.86<br>86–102	89.0<br>±4.22<br>82–95	31.3<br>±1.71<br>29–35	31.1<br>±1.38<br>29–33	41.8<br>±1.40<br>40–44	39.9<br>±1.22<br>37–42	75.2<br>±4.84<br>69.8–85.4	77.9<br>±3.12<br>72.5–82.5

Table S10. Indians' breadths across the nasal saddle, nasal aperture measurements and nasal index – means, standard deviations and ranges

Series	♂ DKB (mm)	♀ DKB (mm)	♂ WNB (mm)	♀ WNB (mm)	♂ NLH (mm)	♀ NLH (mm)	♂ NLB (mm)	♀ NLB (mm)	♂ NLH: NLB	♀ NLH: NLB
Punjabi<br>♂ n=119,<br>♀ n=69–70	20.2<br>±2.46<br>13–29	19.1<br>±2.37<br>16–28	9.3<br>±2.22<br>3–19	8.7<br>±1.53<br>5–13	48.8<br>±3.07<br>42–57	46.3<br>±3.56<br>34–54	25.0<br>±2.20<br>19–32	24.1<br>±2.11<br>19–30	51.2<br>±4.43<br>39.5–63.3	52.2<br>±5.00<br>40.8–64.3
Haryanavi<br>♂ n=96, ♀ n=52	20.0<br>±2.34<br>15–26	19.3<br>±2.03<br>14–25	9.2<br>±1.58<br>5–14	8.9<br>±1.86<br>5–14	50.5<br>±3.19<br>42–59	48.5<br>±4.23<br>34–55	25.5<br>±1.92<br>22–32	24.4<br>±2.21<br>20–29	50.7<br>±4.21<br>39.0–61.4	50.6<br>±6.01<br>38.9–70.6
Hindi<br>♂ n=180–182, ♀ n=120–121	19.6<br>±2.41<br>10–28	18.7<br>±2.24<br>14–25	8.7<br>±1.69<br>5–14	8.6<br>±1.93<br>5–16	49.1<br>±2.88<br>41–59	46.2<br>±3.68<br>36–53	24.8<br>±1.95<br>20–30	23.7<br>±2.07<br>19–28	50.8<br>±4.58<br>39.2–65.9	51.5<br>±4.16<br>40.9–62.2
Urdu<br>♂ n=14, ♀ n=6	18.9<br>±1.77<br>15–21	18.5<br>±1.76<br>16–21	8.9<br>±1.46<br>6–12	8.0<br>±2.28<br>5–10	49.9<br>±3.06<br>41–54	46.2<br>±4.40<br>40–51	25.6<br>±3.37<br>15–29	24.7<br>±1.21<br>23–28	51.2<br>±5.48<br>36.6–58.0	53.9<br>±6.37<br>47.9–61.9
Konkani<br>♂ n=20, ♀ n=4	17.9<br>±2.55<br>9–22	19.5<br>±2.52<br>16–22	8.9<br>±1.48<br>6–12	10.3<br>±4.99<br>6–17	49.4<br>±3.05<br>45–57	48.0<br>±2.94<br>45–51	25.0<br>±2.13<br>21–29	24.2<br>±2.22<br>21–26	50.8<br>±4.63<br>42.3–58.3	50.5<br>±3.16<br>46.7–54.3

Telugu ♂ n=77–79, ♀ n=54–55	18.5 ±2.53 13–26	17.5 ±2.56 10–22	8.6 ±2.00 5–15	8.6 ±1.86 5–13	49.3 ±2.68 42–55	45.5 ±4.30 34–54	25.3 ±1.73 21–29	23.2 ±2.57 15–26	51.4 ±3.98 38.9–61.4	51.3 ±5.59 35.4–62.2
Kannada ♂ n=149, ♀ n=60–62	18.7 ±2.81 11–26	18.0 ±2.42 12–24	9.8 ±2.02 5–15	9.9 ±2.68 5–17	48.0 ±3.06 39–57	45.6 ±2.81 40–52	24.7 ±1.90 20–29	23.7 ±2.32 19–29	51.6 ±4.67 37.7–64.4	52.0 ±5.02 42.2–61.4
Tulu ♂ n=31, ♀ n=18–19	18.9 ±2.89 12–24	17.3 ±2.14 15–24	9.4 ±2.23 6–16	8.5 ±1.39 7–11	49.0 ±2.77 40–53	43.6 ±4.22 34–52	24.9 ±2.96 16–32	22.5 ±2.26 19–26	50.9 ±6.93 33.3–67.5	51.8 ±4.34 43.5–60.5
Tamil ♂ n=114–116, ♀ n=70–71	17.8 ±2.24 11–24	17.3 ±2.42 12–24	8.5 ±1.76 3–14	7.9 ±1.98 4–12	49.0 ±2.78 42–55	45.9 ±3.54 38–57	25.4 ±1.86 21–29	24.2 ±2.42 19–30	52.1 ±5.18 41.8–80.0	52.8 ±4.09 44.4–62.5
Malayalam ♂ n=13, ♀ n=11–12	17.0 ±2.12 14–20	17.3 ±1.92 13–20	8.5 ±1.66 5–10	8.3 ±1.23 7–10	48.9 ±1.75 46–52	45.5 ±3.72 37–50	25.2 ±1.74 23–28	25.2 ±2.59 22–29	51.6 ±3.46 46.0–56.3	54.7 ±3.91 47.8–60.5

Table S11. Indians' mid-facial subtenses (in mm) – means, standard deviations and ranges

Series	♂ DKS (mm)	♀ DKS (mm)	♂ SSS (mm)	♀ SSS (mm)	♂ NDS (mm)	♀ NDS (mm)	♂ SIS (mm)	♀ SIS (mm)	♂ MLS (mm)	♀ MLS (mm)
Punjabi<br>♂ n=117–119, ♀ n=68–70	13.2<br>±2.89<br>6–27	13.4<br>±2.73<br>8–24	24.2<br>±3.04<br>14–31	22.8<br>±3.44<br>15–33	10.5<br>±2.49<br>4–18	9.3<br>±2.31<br>3–15	4.2<br>±1.39<br>1–8	3.5<br>±1.41<br>1–7	9.9<br>±1.65<br>5–13	9.3<br>±1.96<br>3–14
Haryanavi<br>♂ n=96, ♀ n=52	14.3<br>±2.33<br>10–20	13.8<br>±1.98<br>9–19	24.8<br>±2.64<br>17–30	24.3<br>±3.46<br>17–31	12.0<br>±2.31<br>8–19	10.6<br>±1.85<br>7–15	5.3<br>±1.34<br>3–10	4.9<br>±1.33<br>2–8	10.3<br>±1.67<br>7–14	9.8<br>±1.76<br>6–14
Hindi<br>♂ n=180–181, ♀ n= 118–119	13.1<br>±2.07<br>8–19	12.9<br>±2.16<br>6–17	24.2<br>±3.26<br>16–34	22.8<br>±3.26<br>16–33	10.6<br>±2.25<br>4–16	9.1<br>±2.03<br>5–15	4.7<br>±1.36<br>1–9	4.1<br>±1.42<br>2–8	9.8<br>±1.53<br>5–14	9.4<br>±1.67<br>6–17
Urdu<br>♂ n=14, ♀ n=4–6	16.9<br>±2.11<br>13–20	14.7<br>±1.97<br>12–18	23.5<br>±3.20<br>18–29	22.3<br>±2.07<br>20–25	11.1<br>±2.07<br>6–14	8.5<br>±3.21<br>6–13	5.9<br>±1.83<br>2–8	4.0<br>±0.82<br>3–5	10.1<br>±1.56<br>7–12	9.8<br>±2.64<br>7–14
Konkani<br>♂ n=18–20, ♀ n=4	17.5<br>±2.04<br>12–20	15.8<br>±2.99<br>13–20	24.2<br>±4.55<br>14–30	18.5<br>±5.57<br>11–24	9.3<br>±2.55<br>4–13	9.8<br>±1.89<br>7–11	4.6<br>±1.43<br>2–7	3.3<br>±0.50<br>3–4	9.7<br>±1.38<br>6–12	9.3<br>±0.96<br>8–10

Telugu<br>♂ n=76–79, ♀ n=54–55	14.1<br>±2.52<br>8–19	13.9<br>±2.82<br>8–22	23.5<br>±3.07<br>15–31	21.9<br>±2.69<br>17–28	10.3<br>±2.64<br>4–16	8.6<br>±2.55<br>4–14	5.0<br>±1.33<br>2–8	4.0<br>±1.44<br>2–7	9.6<br>±1.71<br>5–13	9.0<br>±2.03<br>5–15
Kannada<br>♂ n=149, ♀ n=60–61	16.1<br>±2.96<br>10–26	15.3<br>±2.81<br>10–25	22.6<br>±3.95<br>12–34	21.6<br>±2.98<br>12–28	8.9<br>±2.41<br>4–15	8.4<br>±2.37<br>4–15	4.0<br>±1.39<br>1–9	3.8<br>±1.39<br>1–10	9.9<br>±1.89<br>4–17	8.7<br>±1.74<br>4–13
Tulu<br>♂ n=31, ♀ n=17–19	17.1<br>±2.94<br>12–23	15.2<br>±3.01<br>8–19	23.5<br>±3.35<br>15–29	20.1<br>±3.41<br>10–24	9.3<br>±2.33<br>5–13	8.5<br>±2.34<br>5–15	4.3<br>±1.62<br>1–8	3.7<br>±1.00<br>2–6	9.9<br>±1.86<br>7–14	9.2<br>±1.32<br>6–11
Tamil<br>♂ n=108–116, ♀ n=68–71	16.0<br>±2.62<br>9–23	14.5<br>±2.19<br>9–19	23.2<br>±3.47<br>12–32	21.6<br>±3.10<br>13–28	9.4<br>±2.20<br>4–15	8.2<br>±2.34<br>3–13	4.7<br>±1.54<br>1–9	3.7<br>±1.31<br>1–7	9.4<br>±1.66<br>5–14	9.1<br>±2.12<br>4–15
Malayalam<br>♂ n=11–13, ♀ n=12	15.2<br>±2.23<br>12–19	15.4<br>±2.35<br>13–20	22.6<br>±2.68<br>19–28	19.8<br>±3.79<br>15–27	8.1<br>±1.61<br>5–11	8.9<br>±2.11<br>6–13	4.8<br>±1.48<br>3–9	4.0<br>±1.13<br>2–6	10.2<br>±2.08<br>8–15	9.7<br>±1.44<br>7–12

Table S12. Indians’ mid-facial flatness indices – means, standard deviations and ranges

Series	♂ EKB: DKS	♀ EKB: DKS	♂ ZMB: SSS	♀ ZMB: SSS	♂ DKB: NDS	♀ DKB: NDS	♂ WNB: SIS	♀ WNB: SIS
Punjabi	14.0	14.7	26.2	25.6	52.6	49.3	46.6	40.9
♂ n=119,	±3.06	±2.93	±3.26	±3.48	±12.82	±12.35	±15.72	±15.47
♀ n=68–	6.1–	8.3–	15.6–	18.1–	17.4–	18.8–	11.1–	10.0–
70	27.6	25.3	34.2	34.4	85.3	88.2	80.0	71.7
Haryanavi	15.2	15.1	27.1	26.9	60.9	55.7	58.6	57.5
♂ n=95–	±2.48	±2.11	±3.09	±3.43	±12.36	±11.21	±15.61	±18.74
96, ♀	10.6–	10.3–	18.7–	18.1–	38.1–	38.1–	27.3–	16.7–
n=52	22.0	20.9	33.3	33.0	95.0	83.3	100.0	100.0
Hindi	14.0	14.3	26.2	25.9	54.7	49.3	55.3	48.8
♂ n=180–	±2.20	±2.37	±3.43	±3.49	±12.54	±11.67	±15.82	±15.72
181, ♀ n=	8.6–	7.1–	17.6–	18.5–	23.3–	25.0–	18.2–	18.8–
118–119	20.0	18.9	34.4	36.3	87.5	85.7	100.0	90.9
Urdu	17.6	15.3	24.9	25.2	59.0	46.3	66.4	53.2
♂ n=14, ♀	±2.15	±1.79	±3.48	±3.16	±10.54	±17.04	±19.66	±18.35
n=4–6	13.7–	12.8–	18.9–	21.3–	33.3–	33.3–	25.0–	40.0–
	20.9	18.0	30.8	30.5	75.0	72.2	100.0	80.0
Konkani	18.3	16.6	25.6	20.5	51.6	49.7	51.8	36.7
♂ n=18–	±2.18	±2.95	±4.59	±5.96	±12.62	±4.61	±16.68	±13.88
20, ♀ n=4	12.5–	14.8–	14.7–	12.0–	33.3–	43.8–	20.0–	17.6–
	22.0	21.1	32.6	24.7	81.3	55.0	87.5	50.0

Telugu	14.8	16.0	25.2	25.0	56.0	48.9	59.8	46.6
♂ n=76–	±2.53	±2.76	±3.15	±2.65	±14.05	±14.05	±16.88	±14.90
79, ♀	8.2–	8.2–	16.3–	19.1–	22.2–	25.0–	25.0–	22.2–
n=54–55	19.6	22.4	32.3	31.0	94.1	86.7	100.0	85.7
Kannada	17.2	17.0	24.5	24.6	47.8	46.8	42.1	39.9
♂ n=149,	±3.08	±3.00	±4.02	±3.44	±12.13	±11.56	±14.94	±12.56
♀ n=60–	10.4–	11.6–	12.9–	13.6–	22.2–	22.2–	11.1-	10.0–
61	27.4	27.2	37.1	31.8	86.7	75.0	87.5	66.7
Tulu	17.9	16.9	25.3	23.5	50.5	49.3	47.4	43.8
♂ n=31, ♀	±3.11	±3.23	±3.72	±4.17	±15.76	±12.98	±18.42	±11.02
n=17–19	12.7–	9.1–	17.0–	12.2–	28.6–	25.0–	9.1–	27.3–
	23.9	20.7	31.5	29.6	86.7	78.9	85.7	71.4
Tamil	17.0	16.1	25.0	24.5	53.1	48.0	55.9	48.7
♂ n=108–	±2.72	±2.39	±3.61	±3.55	±13.76	±14.80	±17.45	±17.50
116, ♀	8.9–	9.5–	13.8–	15.1–	23.5–	16.7–	20.0–	12.5–
n=68–71	23.7	20.5	34.1	31.8	93.8	80.0	100.0	100.0
Malayalam	16.1	16.8	24.7	22.3	48.5	51.9	57.5	48.9
♂ n=11–	±2.48	±2.57	±3.06	±4.63	±12.46	±12.33	±17.93	±16.60
13, ♀	11.9–	13.7–	18.6–	16.1–	25.0–	30.0–	37.5–	28.6–
n=12	19.8	21.3	30.4	29.3	73.3	68.8	90.0	85.7

Table S13. Indians' minimum cranial breadth and lower facial measurements – means, standard deviations and ranges

Series	♂ WCB (mm)	♀ WCB (mm)	♂ MAB (mm)	♀ MAB (mm)	♂ IML (mm)	♀ IML (mm)	♂ XML (mm)	♀ XML (mm)	♂ WMH (mm)	♀ WMH (mm)
Punjabi ♂ n=118–119, ♀ n=68–70	70.5 ±4.29 59–82	68.4 ±4.35 54–78	61.3 ±5.00 47–73	59.3 ±4.65 48–72	32.8 ±3.87 23–42	30.3 ±4.23 21–39	50.0 ±4.76 38–62	46.7 ±4.63 34–56	21.2 ±2.87 11–29	19.1 ±2.48 13–24
Haryanavi ♂ n=96, ♀ n=52	67.8 ±4.92 57–84	64.3 ±5.45 50–79	60.1 ±4.36 48–69	59.8 ±4.47 48–67	33.8 ±3.61 21–41	31.9 ±3.83 24–41	51.5 ±3.55 42–59	48.7 ±4.06 40–57	21.9 ±2.52 14–30	20.8 ±2.25 16–27
Hindi ♂ n=181–182, ♀ n= 118–121	69.7 ±3.70 59–79	65.7 ±4.17 57–76	61.0 ±4.41 48–71	58.4 ±4.04 44–69	32.9 ±3.39 23–41	30.4 ±3.69 23–40	50.2 ±3.83 34–61	46.9 ±3.65 36–55	21.1 ±2.13 16–26	19.7 ±2.23 15–25
Urdu ♂ n=14, ♀ n=6	69.8 ±5.86 55–78	66.5 ±5.13 62–76	62.1 ±4.73 53–71	58.7 ±1.86 57–62	34.6 ±2.59 30–41	31.5 ±6.35 22–39	52.8 ±3.33 45–57	46.7 ±8.69 35–57	22.3 ±2.02 19–25	21.2 ±2.48 18–25
Konkani ♂ n=20, ♀ n=4	70.6 ±4.06 62–78	71.3 ±7.76 65–81	63.3 ±4.15 54–69	58.0 ±2.16 55–60	34.3 ±3.57 26–40	29.3 ±4.03 25–34	51.7 ±3.88 43–58	45.8 ±4.35 40–50	21.2 ±1.67 18–24	20.5 ±1.91 19–23

Telugu ♂ n=78–79, ♀ n=54–55	69.0 ±4.06 58–77	66.4 ±4.73 57–80	61.6 ±4.53 50–70	57.8 ±4.93 46–69	33.3 ±3.59 26–42	30.5 ±4.02 22–39	50.1 ±4.24 41–60	45.7 ±4.93 37–59	21.5 ±2.68 12–27	18.7 ±2.15 14–23
Kannada ♂ n=147–149, ♀ n=61–62	68.8 ±3.89 59–79	65.8 ±4.34 56–75	59.6 ±4.19 45–67	56.7 ±3.83 46–64	32.7 ±3.74 21–42	31.0 ±3.50 20–39	48.8 ±4.23 40–59	45.6 ±3.77 37–53	21.4 ±2.60 16–29	19.7 ±1.90 15–24
Tulu ♂ n=31, ♀ n=18–19	70.2 ±4.33 62–82	65.8 ±3.56 57–73	60.6 ±4.93 52–70	56.6 ±5.47 45–66	34.7 ±3.63 26–42	31.9 ±4.20 25–42	52.0 ±4.05 45–61	46.9 ±4.68 41–59	20.4 ±1.72 17–24	18.3 ±2.05 15–22
Tamil ♂ n=114–116, ♀ n=70–71	69.2 ±3.80 61–81	66.7 ±4.03 60–78	60.6 ±4.88 46–71	56.4 ±4.92 46–68	33.9 ±3.50 27–44	30.9 ±3.61 22–38	49.8 ±4.15 39–61	45.7 ±4.94 33–57	21.1 ±2.06 15–27	19.1 ±2.13 14–25
Malayalam ♂ n=12–13, ♀ n=12	71.1 ±4.29 62–76	68.8 ±4.31 59–74	62.8 ±2.92 58–67	59.3 ±2.45 56–64	35.4 ±4.34 25–40	33.3 ±1.96 30–36	51.6 ±3.80 45–57	47.8 ±3.16 44–53	21.8 ±1.63 18–24	19.1 ±2.31 15–23
